# Targeting of Fzr/Cdh1 for timely activation of the APC/C at the centrosome during mitotic exit

**DOI:** 10.1038/ncomms12607

**Published:** 2016-08-25

**Authors:** Francesco Meghini, Torcato Martins, Xavier Tait, Kazuyuki Fujimitsu, Hiroyuki Yamano, David M. Glover, Yuu Kimata

**Affiliations:** 1 Cell Cycle Development Group, Department of Genetics, University of Cambridge, Downing Street, Cambridge CB2 3EH, UK; 2 Cell Cycle Genetics Group, Department of Genetics, University of Cambridge, Downing Street, Cambridge CB2 3EH, UK; 3 Cell Cycle Control Group, UCL Cancer Institute, University College London, 72 Huntley Street, London WC1E 6DD, UK

## Abstract

A multi-subunit ubiquitin ligase, the anaphase-promoting complex/cyclosome (APC/C), regulates critical cellular processes including the cell cycle. To accomplish its diverse functions, APC/C activity must be precisely regulated in time and space. The interphase APC/C activator Fizzy-related (Fzr or Cdh1) is localized at centrosomes in animal cells. However, neither the mechanism of its localization nor its importance is clear. Here we identify the centrosome component Spd2 as a major partner of Fzr in *Drosophila.* The localization of Fzr to the centriole during interphase depends on direct interaction with Spd2. By generating Spd2 mutants unable to bind Fzr, we show that centrosomal localization of Fzr is essential for optimal APC/C activation towards its centrosomal substrate Aurora A. Finally, we show that Spd2 is also a novel APC/C^Fzr^ substrate. Our study is the first to demonstrate the critical importance of distinct subcellular pools of APC/C activators in the spatiotemporal control of APC/C activity.

## Abstract (web summary)

The activity of the anaphase-promoting complex/cyclosome (APC/C) needs to be regulated in time and space to perform different functions. Here the authors show that Spd2 localizes the APC/C activator Fzr at the centrosomes to promote optimal APC/C activity towards its centrosomal substrate Aurora A.

## Graphical abstract

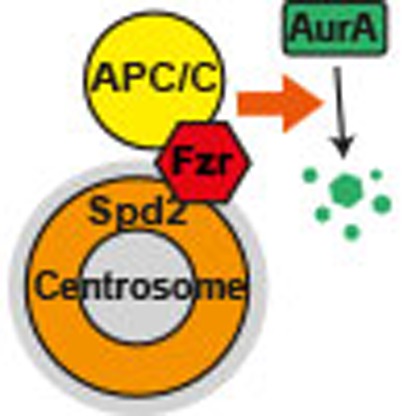

Amulti-subunit ubiquitin ligase, the anaphase-promoting complex or cyclosome (APC/C), controls cell cycle progression through ubiquitin-mediated proteolysis^1^,^2^. By targeting numerous proteins for destruction, the APC/C ensures strict control over the cell cycle. Misregulation of APC/C activity can therefore result in genomic instability, leading to cell death or transformation. Consequently, genes encoding APC/C subunits and its regulators are frequently found to be mutated or amplified in human cancers^3^,^4^. Furthermore, in addition to its established function in cell cycle control, the APC/C is crucial for other aspects of biology in multicellular organisms, such as differentiation, metabolism and brain function^5^. How these diverse functions of the APC/C are spatiotemporally regulated and mutually coordinated remains elusive^6^.

The CDC20 family of APC/C activator proteins constitute the primary group of APC/C regulatory proteins^7^. These activators share two distinct and complementary protein domains that are important for the APC/C-dependent ubiquitination reaction: the WD40 repeat domain supports substrate interactions, whilst the N-terminal domain containing the C-box motif stimulates APC/C's catalytic activity^8^,^9^. The current model for the regulation of APC/C activity is based solely on its sequential interaction with the activators: Fizzy (Fzy, also known as CDC20) and Fizzy-related (Fzr, also known as Cdh1)^1^,^7^. Fzy binds and activates the APC/C in early mitosis to trigger chromatid separation and cyclin degradation. Following the inactivation of cyclin-dependent kinase 1 (Cdk1), Fzr interacts with the APC/C to maintain its activity throughout G1 phase. However, this simplistic model cannot accommodate the expanding repertoire of APC/C functions in metazoans. It is unable to explain how the APC/C can target a vast number of substrates in a strict spatiotemporal order, some of which localize to distinct cellular compartments during specific time windows. Nor can it explain how the APC/C coordinates its cell cycle functions with its other key functions in differentiating and terminally differentiated tissues.

Spatial regulation might confer an additional dimension to the control of the multitude of APC/C functions^6^,^10^. Strong correlations between the subcellular localization of APC/C activators and the functional states of the APC/C have been observed. In early mitosis, the accumulation of Fzy at unattached kinetochores correlates with the inactive state of the APC/C (ref. 11). In postmitotic neurons in the mammalian brain, Fzy is localized at centrosomes to specifically regulate dendrite morphology, whereas Fzr accumulates in the nucleus to modulate axonal growth^12^,^13^. These observations point to the regulation of spatially distinct APC/C pools through the localization of APC/C activators. Since Fzy has emerged as a potent anti-cancer target^14^ and Fzr is a haploinsufficient tumour suppressor^15^, understanding how these two activators control APC/C in space and time is crucial for clarifying the role of the APC/C in cancer.

APC/C components and regulators are highly enriched at the centrosomes in a variety of metazoan cells, highlighting the potential function of this organelle as a control hub for the APC/C (refs 13, 16, 17, 18, 19, 20). The centrosome is a major microtubule-organising centre comprising of a pair of cylindrical tubular structures, the centrioles and a surrounding proteinaceous matrix, the pericentriolar material (PCM)^21^. The centrosome regulates division, polarization and migration of animal cells and its dysregulation is prevalent in cancer and several genetic disorders^22^. In *Drosophila* embryos and human cells, the degradation of the canonical APC/C substrate, Cyclin B (CycB), begins at centrosomes and mitotic spindles on anaphase onset (AO)^23^,^24^. This, in combination with the dynamic localization of Fzy and Fzr to centrosomes, strongly suggests that their centrosomal localization may be crucial for the spatiotemporal regulation of APC/C activity^16^,^17^. However, this model has not been tested because of an inability to specifically manipulate centrosome-associated pools of Fzy or Fzr.

In this study, we investigate the centrosome-specific localization and function of the APC/C activator, Fzr, in *Drosophila melanogaster*. We show that Fzr localizes to centrosomes in a cell cycle-dependent manner. Mass spectrometric analyses of APC/C-interacting proteins identified a core centrosome component, Spd2, as the centrosomal receptor for Fzr. By creating Spd2 mutants that specifically alter Fzr binding, we have uncovered a specific role for the centrosomal pool of Fzr in the regulation of APC/C-dependent proteolysis during mitotic exit in neural stem cells. Finally, we also show that Spd2 is targeted by APC/C^Fzr^ for degradation, pointing to a potential negative feedback loop between Spd2 and Fzr at the centrosome.

## Results

### Fzr localizes to the centriole

**Figure 1:**
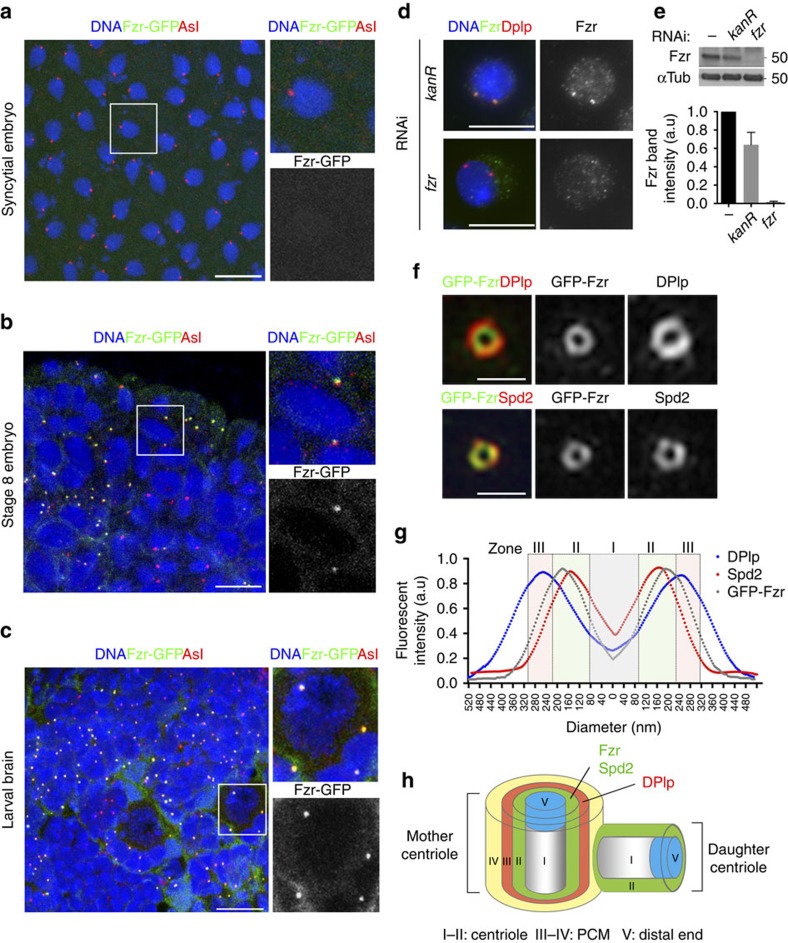
Endogenous Fzr coincides with Spd2 in the inner centriole. (**a**) *Drosophila* syncytial embryos carrying the *fzr-GFP*^*fosmid*^ stained for DNA (blue) and centrosome marker Asl (red). The inset of an individual nucleus shows that there is no clear Fzr-GFP (green) at the centrosomes. (**b**) Fzr-GFP^fosmid^ expressing stage eight embryo stained for DNA (blue) and centrosome marker Asl (red), showing Fzr-GFP (green) localization at the centrosomes. (**c**) NBs of third instar larvae carrying the *fzr-GFP*^*fosmid*^ showing the centrosomal localization of Fzr-GFP (green). The inset of an individual NB shows the co-localization of Fzr-GFP with the centriole marker Asl (red). (**d**) Cultured *D. mel-2* cells stained for DNA (blue), endogenous Fzr (green) and a centrosomal marker DPlp (red) following pre-extraction of the cytoplasm. Centrosomal localization of Fzr was observed in control cells (*kanR* RNAi, top), but not in Fzr-depleted cells (*fzr* RNAi. bottom). (**e**) Immunoblots of the *D. mel-2* cell extracts confirming the depletion of endogenous Fzr following *fzr* RNAi, but not *kanR* RNAi (top). The relative Fzr band intensity was quantified and the mean values are indicated in the bar graph (bottom). *n*=2. Error bars: s.d. (**f**) 3D-SIM super-resolution images of interphase centrosomes in *D. mel-2* cells expressing GFP-Fzr (green) stained for DPlp (red in the top panels) or Spd2 (red in the bottom panels). GFP-Fzr co-localizes with Spd2 at the inner region of the centriole within a ring formed by DPlp. (**g**) Distribution curves of the signal intensities of GFP-Fzr, Spd2 and DPlp signals along the diameter of centrioles. The *x* axis indicates the distance from the centre of the centriole (nm). The plot of the signal intensity of GFP-Fzr and Spd2 show the co-localization (overlapping peaks) inside of the DPlp peaks. (**h**) A schematic view of the multi-layered structure of the *Drosophila* centrosome. Fzr and Spd2 coincide in ‘Zone II' within a mother centriole. Scale bars correspond to 10 μm, except **f** where the scale bars correspond to 300 nm.

It was previously shown that exogenous green fluorescent protein (GFP)-tagged Fzr (GFP-Fzr) localizes to the centrosomes in *Drosophila* syncytial blastoderm embryos^16^. However, endogenous Fzr is not expressed at this early developmental stage^16^,^25^. To clarify the subcellular localization of Fzr expressed at its endogenous levels, we first examined a fly line expressing Fzr fused to a 2xTY1-GFP-V5 tag under its endogenous promoter (*fzr-GFP*^*fosmid*^)^26^. We confirmed the expression level of Fzr-GFP^fosmid^ to be comparable to endogenous Fzr in larval brain extracts (Supplementary Fig. 1a,b). The *fzr-GFP*^*fosmid*^ fully rescued the lethality of a *fzr*-null allele, *fzr*^*ie28*^ (ref. 27). In accordance with previous studies, no Fzr-GFP^fosmid^ signal was detected in the syncytial blastoderm (Fig. 1a). A weak cytoplasmic GFP signal appeared on cellularization, and clear punctate GFP signals co-localized with the centrosome marker, Asterless (Asl), in the embryos in stage eight onwards (Fig. 1b). Distinct centrosomal Fzr-GFP^fosmid^ was also observed in various postembryonic tissues, including neural stem cells, neuroblasts (NBs), in the larval central nervous system (Fig. 1c) and epithelial follicle cells in the egg chamber of adult females (Supplementary Fig. 2a), the two tissues that highly express *fzr* messenger RNA (ref. 28). We also observed endogenous Fzr at centrosomes in cultured *D. mel-2* cells using a Fzr-specific antibody^16^. Pre-extraction of the cytoplasm allowed us to observe distinct centrosomal Fzr, which was abolished on Fzr depletion by RNA interference (RNAi, Fig. 1d,e). These data strongly suggest that endogenous Fzr is localized at centrosomes.

Subdiffraction-resolution microscopic techniques have revealed a concentric multilayer structure within the interphase centrosome in *Drosophila* and human cells^29^,^30^,^31^,^32^. To determine the precise location of Fzr within the centrosome, we performed 3D-structured illumination microscopy (3D-SIM) on Fzr in cultured *D. mel-2* cells. We introduced GFP- or FLAG-tagged Fzr into these cells and confirmed that both fusion proteins clearly localized to centrosomes during interphase (Supplementary Fig. 3a–c). We observed a ring-like organization of GFP-Fzr coincident with a known centrosome component, Spd2, in an inner region of the interphase centriole, which we previously termed ‘Zone II'^29^,^30^,^31^,^32^ that lies inside the ‘Zone III' ring marked by the pericentrin-like protein DPlp (Fig. 1f–h). We also analysed the localization of the mitotic APC/C activator Fzy within the centrosome and found that GFP-Fzy co-localized with GFP-Fzr in ‘Zone II' (Supplementary Fig. 4a–c). We conclude that *Drosophila* Fzr and Fzy specifically localize to ‘Zone II' within the centriole.

### The cell cycle-dependent centrosomal localization of Fzr

**Figure 2:**
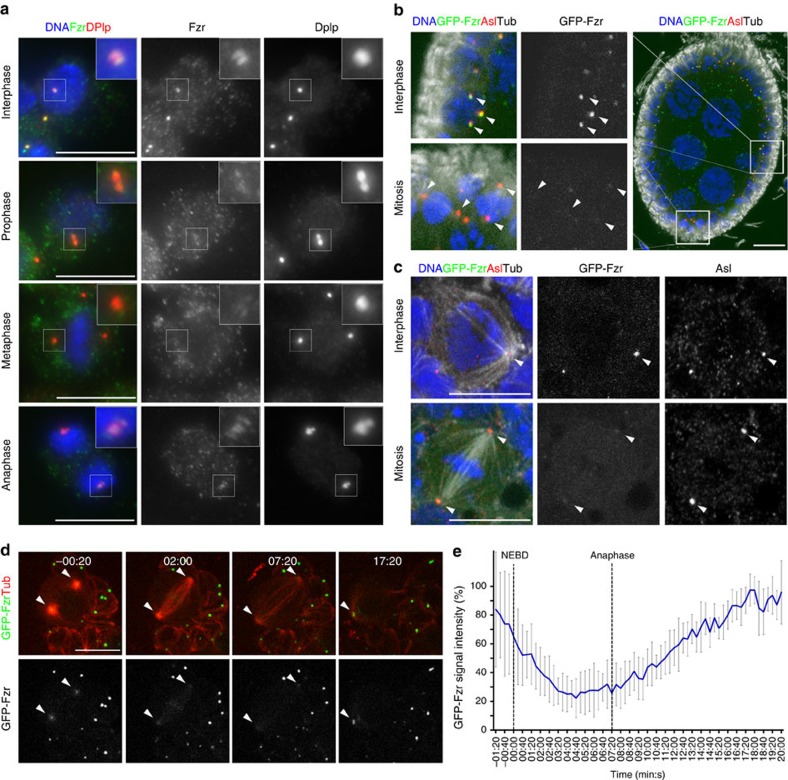
Fzr association with the centrosome is regulated during the cell cycle. (**a**) *D. mel-2* cells were fixed after pre-extraction and stained with a Fzr-specific antibody (green), DNA (blue) and the centrosome marker DPlp (red). During interphase (top panels), there were strong Fzr signals at the centrosomes, co-localizing with DPlp (red). After mitotic entry, the centrosomal level of Fzr drastically decreased (prophase and metaphase, the second and third top panels) and only began to reappear during late anaphase (bottom panels). (**b**) A *Drosophila* egg chamber expressing GFP-Fzr (green), and stained for Asl (red) and α-Tubulin (Tub, white). Interphase follicle cells showed clear centrosomal GFP-Fzr (top left panels), whereas in mitotic follicle cells GFP-Fzr was mostly absent at the centrosomes (bottom left panels). White arrowheads point to the centrosomes. (**c**) Third instar larval NBs expressing GFP-Fzr (green) and stained for DNA (blue) and Asl (red) show a similar cell cycle-dependent oscillation of centrosomal GFP-Fzr: GFP-Fzr is present at the centrosome in interphase cells (top panel) but it dissociates from centrosomes in mitotic NBs (bottom panel). White arrows point to the centrosomes. (**d**) Tiles of selected still images from time-lapse live imaging of *Drosophila* NBs co-expressing GFP-Fzr (green) and mCherry-Tubulin (Tub, red) driven by wor-Gal4 driver, showing the dynamics of Fzr localization during mitosis. Centrosomal GFP-Fzr levels significantly decreased after mitotic entry, reached a minimum at metaphase and re-accumulated after anaphase. The white arrows indicate the centrosomes. (**e**) Centrosomal GFP-Fzr fluorescence intensities in the NBs were measured at each time point and are presented in the line graph (*n*=8, error bars indicate s.d.). Vertical dotted lines highlight the nuclear envelope break down (NEBD; used as time reference, 00:00) and anaphase (average time 07:20 after NEBD). Scale bars correspond to 10 μm, except **d** where the scale bars correspond to 5 μm.

In *Drosophila* syncytial embryos GFP-Fzr, expressed under a constitutively active polyubiquitin promoter, localize to centrosomes at all stages of the nuclear division cycle^16^. However, these cycles are highly unusual because of their rapidity, lack of gap phases and the absence of cytokinesis. We therefore examined Fzr localization in *D. mel-2* cells, larval NBs and follicular epithelial cells, which undergo the conventional four-phase cell cycle. In all these cell types, centrosomal GFP-Fzr was barely detectable during mitosis, in comparison to its robust signal during interphase (Fig. 2a–c). Cell cycle-dependent oscillation of the centrosomal localization of Fzr was confirmed by time-lapse live imaging of larval NBs co-expressing GFP-Fzr and mCherry-Tubulin (Fig. 2d,e, Supplementary Movie 1). Centrosomal GFP-Fzr remained at similar signal intensity during interphase and prophase (−0:20 min, Fig. 2d,e). After nuclear envelope break down, centrosomal GFP-Fzr began to decline reaching its minimal intensity by metaphase (04:00 min, Fig. 2e). After AO, GFP-Fzr slowly re-accumulated at centrosomes and returned to interphase levels during telophase (16:00 min, Fig. 2e). Based on these results, we conclude that Fzr is associated with centrosomes throughout interphase but dissociates from the centrosome during mitosis.

We also investigated the cell cycle-dependent oscillation of Fzy. Similar to GFP-Fzr, GFP-Fzy showed centrosomal localization during interphase and dissociated from centrosomes during mitosis in *D. mel-2* cells and NBs (Supplementary Fig. 4d–g, Supplementary Movie 2). However, GFP-Fzy exhibits a higher cytoplasmic signal throughout the cell cycle and accumulates to very high levels at the kinetochore during prometaphase, occasionally obscuring its centrosomal signals (Supplementary Fig. 4f, Supplementary Movie 2). Live imaging analysis of NBs showed that the centrosomal GFP-Fzy signal started to decline on mitotic entry concurrently with the decrease in centrosomal GFP-Fzr. However, GFP-Fzy remained dissociated and did not return to the centrosome even after GFP-Fzr had returned to its interphase level during mitotic exit (Supplementary Fig. 4f,g). This suggests differential regulation of the centrosomal localization of Fzr and Fzy during the cell cycle.

### Fzr interacts with the centrosome component Spd2

**Figure 3:**
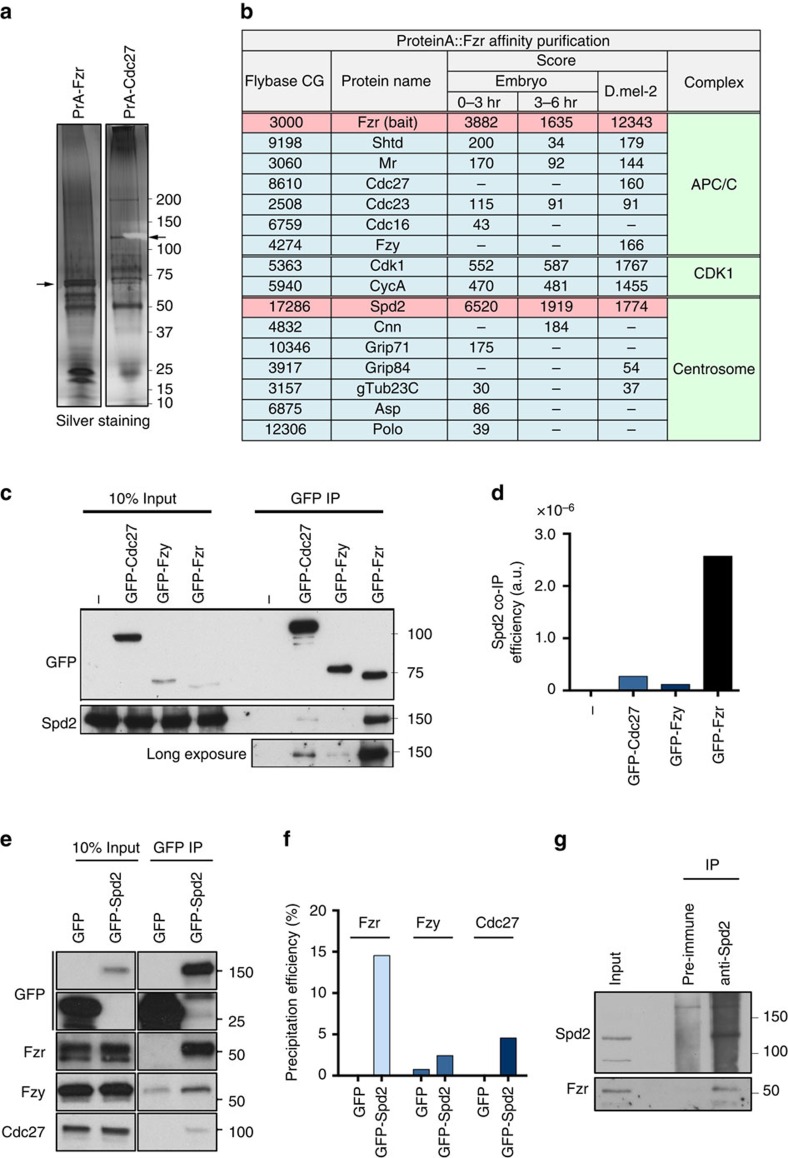
The APC/C coactivator Fzr forms a complex with Spd2 in *Drosophila* cultured cells and embryos. (**a**) PrA-Fzr and PrA-Cdc27 were purified from stably expressing *D. mel-2* cells, and the co-purified proteins were resolved on SDS–PAGE and visualized with silver staining. The bands corresponding to the bait proteins are indicated by the arrows; the other bands represent co-purified proteins. (**b**) List of selected proteins that were co-purified with PrA-Fzr from *D. mel-2* cells, or *Drosophila* embryos collected in the indicated time periods after laying. The listed proteins are categorized according to the protein complexes or organelles that they are known to belong to: the APC/C, Cdk1 and the centrosome. Amongst the potential centrosomal Fzr partners, Spd2 showed significantly high MASCOT score in every sample. (**c**,**d**) Anti-GFP immunoprecipitation from untransfected *D. mel-2* cells (−) or from stable cell lines expressing GFP-Cdc27, GFP-Fzy or GFP-Fzr. The inputs and the precipitates were analysed by immunoblotting against GFP and Spd2. The Spd2 co-IP efficiency of each bait protein was quantified and are shown in the bar graph in **d**. Spd2 was most efficiently co-precipitated with GFP-Fzr. (**e**,**f**) Anti-GFP immunoprecipitation from *D. mel-2* cells stably expressing GFP alone or GFP-tagged Spd2. The inputs and precipitates were blotted against Fzr, Fzy and Cdc27. The Fzr, Fzy or Cdc27 precipitation efficiencies of GFP or GFP-Spd2 were quantified and the values are shown in the bar graph in **f**. Endogenous Fzr was efficiently co-immunoprecipitated with GFP-Spd2, but not GFP alone. (**g**) Immunoprecipitation from wild-type *D. mel-2* cells using an anti-Spd2 antibody, or a pre-immune rabbit serum as control. Endogenous Fzr was specifically co-immunoprecipitated with Spd2, demonstrating that endogenous Fzr and Spd2 form a complex.

We next sought to identify the protein responsible for the centrosomal recruitment of Fzr. To this end, we expressed Protein A-tagged Fzr (PrA-Fzr) in both *D. mel-2* cells and early stage embryos and purified the PrA-Fzr complex from the lysates. The co-purified proteins were then analysed by mass spectrometry (MS; Methods section). The Fzr precipitates exhibited several specific bands that were absent in the samples that used the APC/C subunit Cdc27 as bait (Fig. 3a). Mass spectrometric analysis in the PrA-Fzr samples identified many known Fzr interactors, including APC/C subunits and Cdk1, suggesting that PrA-Fzr had been assembled into the endogenous APC/C (Fig. 3b). Moreover, we identified several centrosome components that have never been reported to interact with Fzr or the APC/C (Fig. 3b). In particular, a core centrosome component, Spd2 (refs 33, 34), was repeatedly detected with high Mascot scores.

To confirm the physical interaction between Spd2 and Fzr, we performed co-immunoprecipitation (co-IP) experiments from cultured *D. mel-2* cells as well as *Drosophila* tissues. We generated *D. mel-2* cell lines that stably express GFP-Fzr, GFP-Fzy or GFP-Cdc27. Endogenous Spd2 was efficiently co-precipitated with GFP-Fzr (co-IP efficiency: 2.57 × 10^−6^, Fig. 3c,d). In contrast, a considerably lower amount of Spd2 was co-precipitated with GFP-Cdc27 or GFP-Fzy (co-IP efficiency: 0.27 × 10^−6^ or 0.1 × 10^−6^, respectively, Fig. 3c,d). Consistently, in the reciprocal co-IP, a large proportion of endogenous Fzr co-precipitated with GFP-Spd2 (14.54%, Fig. 3e,f), whilst small quantities of endogenous Fzy or Cdc27 were pulled down with GFP-Spd2 (2.43% or 4.55%, respectively, Fig. 3e,f). Finally, using the Spd2 antibody, we were able to co-immunoprecipitate endogenous Fzr (Fig. 3g). We also detected an interaction between Fzr-GFP^fosmid^ and endogenous Spd2 in the larval brain extracts from the *fzr-GFP*^*fosmid*^ line (Supplementary Fig. 1c). We therefore conclude that the centrosomal component Spd2 is a major interactor of the APC/C activator Fzr in *Drosophila*.

We also tested whether an analogous physical interaction can be observed between Cdh1 and the Spd2 orthologue, Cep192, in human cells. We found that, similar to Fzr, GFP-tagged Cdh1 localized to the centrosome during interphase but not during mitosis in HeLa cells (Supplementary Fig. 5a–c). However, we were unable to detect a physical interaction between Cep192 and Cdh1 in co-IP experiments (Supplementary Fig. 5d,e).

### Fzr binds Spd2 through APC/C degron motifs

**Figure 4:**
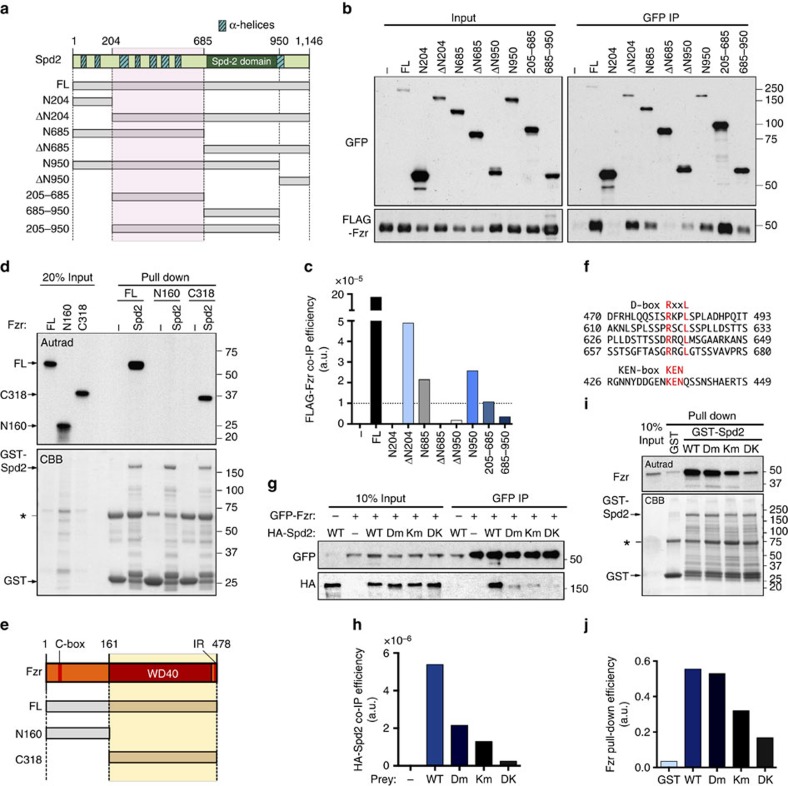
Fzr interacts directly with the middle region of Spd2 via APC/C degron-like motifs. (**a**) Diagram of the truncated forms of Spd2 generated for this study. The Spd2 domain and predicted α-helices (striped boxes) are indicated and the 205–685 amino acid region, mainly responsible for Fzr binding, highlighted in pink. (**b**,**c**) Anti-GFP immunoprecipitation from *D. mel-2* cells co-expressing FLAG-Fzr and various Spd2 fragments fused to GFP. The FLAG-Fzr co-IP efficiency of each fragment was quantified and is indicated in **c**. The Spd2 fragments containing the 205–685 region efficiently co-precipitated FLAG-Fzr. The dotted line indicates the cut-off value of 1.0 e^−5^. (**d**) The *in vitro* binding assay using recombinant GST-Spd2 immobilized on Glutathione Sepharose beads, alongside ^35^S-labelled full length (FL) or the two truncated forms (N160 and C318) of Fzr. FL and C318, but not N160, were pulled down by GST-Spd2. (**e**) Diagram showing the domain organization of Fzr and the truncated forms used in **d**. FzrN160 contains the C-box motif, whereas FzrC318 contains the WD40 repeat domain and the IR motif. (**f**) The amino acid sequence of Spd2 surrounding the four D-box motifs and the KEN-box in the 205–685 region, which are highly conserved in *Drosophila* species. The consensus residues of D-box (RxxL) and KEN-box (KEN) are indicated in red. (**g**,**h**) Anti-GFP immunoprecipitation from *D. mel-2* cells stably expressing HA-Spd2-WT, Dm, Km and DK, alongside inducible GFP-Fzr (induced, +, or uninduced, −). The co-IP efficiencies of GFP-Fzr with the various HA-Spd2 forms were quantified and are shown in **h**. The interaction of Spd2 with Fzr was significantly reduced by the mutation of the D-boxes (Spd2-Dm) or KEN box (Spd2-Km), and completely abolished by the mutation of both motifs (Spd2-DK). (**i**,**j**) The *in vitro* binding assay using purified GST or GST-fused Spd2-WT, Dm, Km and DK alongside ^35^S-labelled Fzr. The Fzr pull-down efficiencies for each bait are shown in **j**. Spd2-Km and Spd2-Dm showed a partial reduction in Fzr binding, and Spd2-DK further reduced the binding capacity for Fzr.

To determine the binding interface between Fzr and Spd2, we generated a series of truncated forms of Spd2 by dividing it into four segments: residues 1–204 with two predicted coiled-coil domains; residues 205–685 with multiple clusters of α-helices and intervening unstructured regions; residues 686–950 containing the conserved Spd2 domain; and the remaining carboxyl terminal segment (residues 951–1,146, Fig. 4a). We introduced these fragments into *D.mel*-2 cells co-expressing FLAG-Fzr and performed co-IP assays. The fragments that lack residues 205–685 showed a significant reduction in their ability to co-precipitate FLAG-Fzr, whereas the fragments containing this region were able to more efficiently co-precipitate Fzr (co-IP efficiency: >1.0 × 10^−5^, Fig. 4b,c).

To determine if Spd2 interacts directly with Fzr, we purified recombinant GST-tagged Spd2 expressed in *E. coli* and tested if it bound ^35^S-methionine-labelled Fzr protein, synthesized by *in vitro* transcription–translation in reticulocyte lysates. GST-Spd2, but not GST alone, efficiently pulled down the recombinant Fzr protein, demonstrating a direct physical interaction between Fzr and Spd2 (Fig. 4d). In accordance with our co-IP results, Fzr strongly interacted with the Spd2 fragments containing the 205–685 segment (Supplementary Fig. 6a,b). We also synthesized two fragments of Fzr: FzrN160, which contains the C-box motif (DRFIP) required for direct interaction with the APC/C and stimulation of its ligase activity^8^, and FzrC318, which possesses the WD40 repeat domain and the IR motif (Fig. 4e). The recombinant GST-Spd2 showed strong affinity for FzrC318, but no interaction with FzrN160 (Fig. 4d). Together, these data indicate that the C-terminal part of Fzr directly interacts with the 205–685 amino acid segment of Spd2.

The WD40 repeat domain is conserved amongst all the known CDC20 family members and interacts directly with the APC/C degron motifs, destruction-box (D-box) and KEN-box^9^,^35^,^36^. Spd2 has highly conserved putative KEN-box and four D-box consensus sequences within its Fzr-interacting domain (Fig. 4f). We created mutant forms of Spd2 that carried mutations either in the KEN-box (Spd2-Km, KEN to AAA) or in the four D-boxes (Spd2-Dm, RxxL to AxxA), or in all the five motifs (Spd2-DK), and performed co-IP experiments in *D. mel-2* cells co-expressing these Spd2 variants fused to a HA tag alongside GFP-Fzr. Both Spd2-Km and Spd2-Dm mutant proteins showed a significant reduction in Fzr interaction when compared with wild-type Spd2, and the Spd2-DK mutant protein failed to interact with Fzr (Fig. 4g,h). We also performed direct binding assays *in vitro* and found that Spd2-Km significantly reduced the ability to bind Fzr. The Dm mutation alone had a minimal effect on Fzr binding in this assay. However, when it was combined with the Km mutation, the resulting Spd2-DK protein showed a further reduction in its interaction with Fzr compared with Spd2-Km (Fig. 4i,j). We also found the mitotic activator Fzy interacted with GST-Spd2 in the *in vitro* binding assay. However, neither the D-box nor the KEN-box mutation in Spd2 affected its interaction with Fzy (Supplementary Fig. 7a,b). Together, these results suggest that the KEN-box and D-box motifs within the 205–685 segment of Spd2 synergistically mediate direct interaction with the WD40 repeat domain of Fzr.

### Spd2 links Fzr to the centrosome

**Figure 5:**
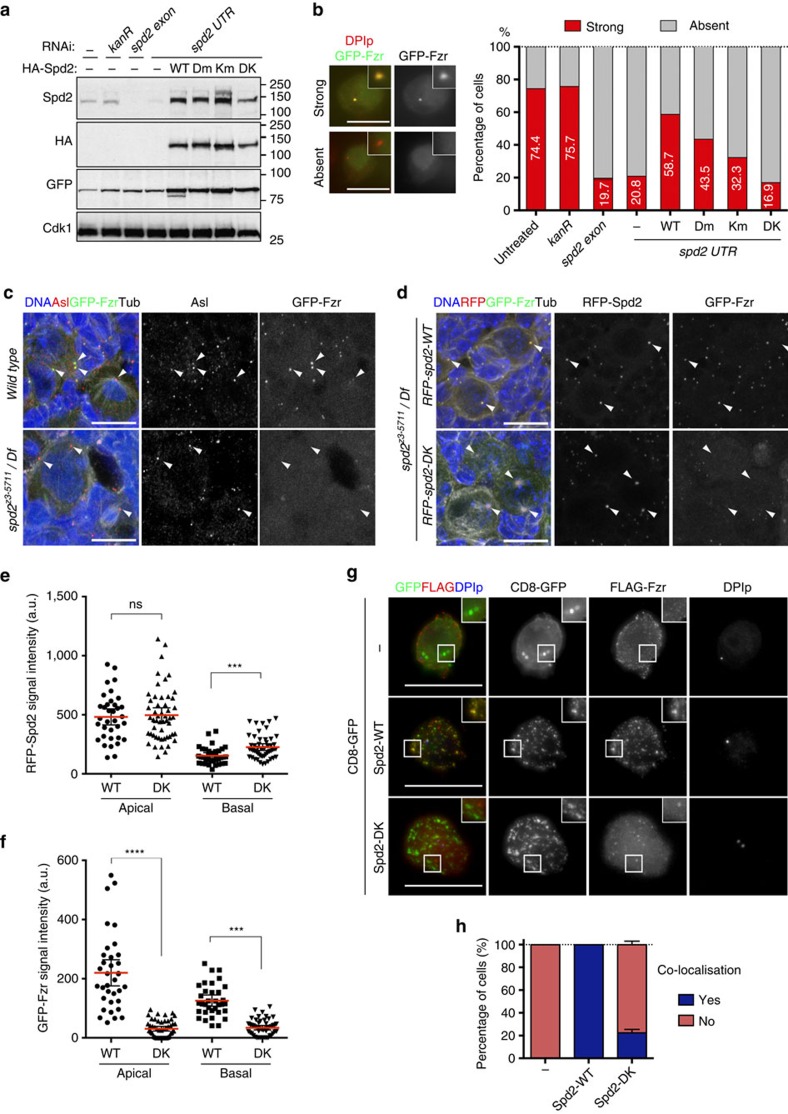
Spd2 is the centrosomal loading factor of Fzr. (**a**) Immunoblotting of cell extracts from *D. mel-2* cells expressing GFP-Fzr alone or in combination with HA-Spd2-WT, Spd2-Dm, Spd2-Km or Spd2-DK. Endogenous Spd2 was depleted by both *spd2 exon* and *spd2 UTR* RNAi. All the HA-Spd2 proteins were expressed at comparable levels. (**b**) The cells used in **a** were stained for DPlp (red) and categorized into two groups: cells displaying centrosomal GFP-Fzr (green; Strong) or cells with no centrosomal GFP-Fzr (Absent). Representative images are shown (left) and the categorization result was shown in the bar graph (right) (*n*=300). Both *spd2 exon* and *spd2 UTR* RNAi significantly reduced the cells exhibiting centrosomal GFP-Fzr. HA-Spd2-WT co-expression restored centrosomal GFP-Fzr, whilst HA-Spd2-DK co-expression showed no rescue. HA-Spd2-Dm or HA-Spd2-Km partially rescued centrosomal GFP-Fzr. (**c**) Wild type or the *spd2*-null mutant NBs expressing GFP-Fzr (green), stained for Asl (red), DNA (blue) and α-Tubulin (white). GFP-Fzr was undetectable at the centrosome in *spd2*-null NBs (bottom panels). The white arrowheads point to the centrosomes. (**d**) The Spd2-WT or Spd2-DK rescued NBs expressing GFP-Fzr (green). Both RFP-Spd2-WT and RFP-Spd2-DK localized at the centrosome. Whilst RFP-Spd2-WT restored the centrosomal localization of GFP-Fzr, RFP-Spd2-DK could not. The white arrows point to the centrosomes. The centrosomal signals of RFP-Spd2 (**e**) or GFP-Fzr (**f**) were quantified in interphase Spd2-WT or Spd2-DK rescued NBs and were plotted in dot blots. The red lines indicate means and error bars 95% CIs. Despite the comparable levels of centrosomal RFP-Spd2-WT and RFP-Spd2-DK (ns, not significant, *P*=0.911, ***P=0.0002, Mann–Whitney *U* test), centrosomal GFP-Fzr was significantly reduced at both apical and basal centrosomes in the Spd2-DK rescued NBs (****P<0.0001, unpaired *t*-test). (**g**) Membrane tethering of Spd2 by fusing a membrane protein, CD8. On co-transfection with CD8-GFP, or CD8-GFP-fused Spd2-WT or Spd2-DK, FLAG-Fzr co-localized with CD8-GFP-Spd2-WT at the ectopic cytoplasmic foci, but not with CD8-GFP-Spd2-DK or CD8-GFP. The insets show the membrane or punctate cytoplasmic localization of the CD8-GFP fusions. (**h**) Quantification of the co-localization of FLAG-Fzr with CD8-GFP, CD8-GFP-Spd2-WT or Spd2-DK at the ectopic cytoplasmic punctate structures. *n*=300. Error bars, s.d.; scale bars, 10 μm.

Spd2 exists in two distinct pools in the centrosome: the PCM pool that accumulates around centrioles during mitosis to recruit other PCM components and nucleate microtubules^30^,^33^,^34^, and the ‘Zone II' centriolar pool that we found to co-localize with Fzr during interphase (Fig. 1f). To determine whether Spd2 is responsible for the localization of Fzr to interphase centrioles, we first examined the effect of Spd2 depletion on the centrosomal localization of GFP-Fzr in *D. mel-2* cells. In the cells stably expressing GFP-Fzr, Spd2 depletion by RNAi against a *spd2* exon (*spd2 exon*) significantly reduced the number of cells exhibiting a clear centrosomal GFP-Fzr signal in comparison to untreated cells or the control RNAi against *kanR* (Fig. 5a,b). We then performed rescue experiments by generating *D. mel-2* cell lines that co-express GFP-Fzr with HA-tagged Spd2-WT, Spd2-Dm, Spd2-Km and Spd2-DK. All of the HA-Spd2 fusions were expressed at comparable levels and accumulated at centrosomes to similar extents (Fig. 5a, Supplementary Fig. 8a,b). Using 3D-SIM, we also confirmed that both GFP-tagged Spd2-WT and Spd2-DK localized to ‘Zone II' in the interphase centriole (Supplementary Fig. 8c,d). When performing the rescue experiments, we used RNAi against an untranslated region of Spd2 (*spd2 UTR*) to specifically deplete endogenous Spd2, but not exogenous HA-Spd2. The *spd2 UTR* RNAi led to efficient depletion of endogenous Spd2 as well as a reduction in the number of cells exhibiting the centrosomal localization of GFP-Fzr, similar to the *spd2 exon* RNAi (Fig. 5a,b). Expression of HA-Spd2-WT in *spd2 UTR* dsRNA-treated cells efficiently restored the centrosomal GFP-Fzr localization (Fig. 5b). In contrast, expression of HA-Spd2-DK showed virtually no rescue of the centrosomal GFP-Fzr recruitment, whereas HA-Spd2-Dm or HA-Spd2-Km expression partially rescued GFP-Fzr localization (Fig. 5b). This suggests that Spd2 recruits Fzr to the centriole through the direct interaction with its KEN-box and D-box motifs.

To confirm the requirement of Spd2 for the centrosomal localization of Fzr *in vivo*, we first examined GFP-Fzr localization in *spd2*-null mutants. Homozygous *spd2*-null mutants are viable and morphologically wild type, however, both sexes are sterile^33^,^34^. When we examined larval NBs in a *spd2*-null mutant (*spd2*^*z3*−*5711*^*/Df(3L)BSC561*) expressing GFP-Fzr, we were unable to detect GFP-Fzr at the centrosome (Fig. 5c). We also observed delocalization of GFP-Fzr in ovarian follicle cells in the *spd2* mutant adult females (Supplementary Fig. 2b). However, we noticed that, although *Drosophila* Spd2 is not required for the duplication of centrioles^33^,^37^, a large number of cells in the *spd2*-null mutants lacked centrioles (judged by the absence of the centriole protein Asl, Fig. 5c, Supplementary Fig. 2b), most likely due to mis-segregation of inactive centrosomes in the preceding mitoses. To rule out the possibility that the apparent Fzr mislocalisation may be indirectly caused by a loss of functional centrosomes, we created fly lines expressing red fluorescence protein (RFP)-tagged Spd2-WT or Spd2-DK. We found that both fusion proteins localized to centrosomes at comparable levels and successfully restored the recruitment of γ-Tubulin and Centrosomin (Cnn) during mitosis in *spd2*-null mutant NBs (Supplementary Fig. 9a–d). The *spd2*-null NBs expressing either RFP-Spd2-WT or RFP-Spd2-DK also efficiently formed bipolar spindles and completed asymmetric cell divisions without any recognizable defects (Supplementary Movies 3 and 4). Moreover, both transgenes fully restored fertility in *spd2*-null mutant flies, suggesting that the Spd2-DK mutant protein is able to fulfil all of the known mitotic functions of Spd2. Nevertheless, whilst RFP-Spd2-WT rescued the centrosomal localization of GFP-Fzr in the *spd2*-null mutant NBs, RFP-Spd2-DK could not recruit GFP-Fzr to centrosomes (Fig. 5d–f). These data strongly suggest that direct physical interaction between Spd2 and Fzr is required for the centrosomal localization of Fzr *in vivo*.

Finally, we examined whether Spd2 is capable of recruiting Fzr to an ectopic cellular location independent of the centrosome. We fused Spd2-WT or Spd2-DK with a plasma membrane component, the human T-cell receptor CD8, along with GFP (ref. 38) and co-expressed FLAG-Fzr with these fusion proteins or with CD8-GFP in *D. mel-2* cells (Supplementary Fig. 10). CD8-GFP accumulated at the plasma membrane and in cytoplasmic vesicles, whilst CD8-GFP-Spd2 fusions formed more punctate vesicles in the cytoplasm (Fig. 5g). We found that FLAG-Fzr was recruited to these ectopic cytoplasmic punctate structures in all the cells expressing CD8-GFP-Spd2-WT observed (Fig. 5g,h). In contrast, only a small proportion of the cells expressing CD8-GFP-Spd2-DK showed the ectopic localization of FLAG-Fzr to GFP (mean±s.d.: 22.33±3.05%, Fig. 5g,h). The expression of CD8-GFP did not affect the localization of FLAG-Fzr. Taken together, these results establish Spd2 as the centrosomal linker for Fzr in *Drosophila*. Spd2 possesses two distinct properties: first, the ability to recruit other PCM components to nucleate microtubules during mitosis, and, second, the ability to recruit Fzr to inner centrioles during interphase, which requires its KEN- and D-box motifs.

### Centrosomal Fzr is required for timely Aurora A destruction

The above findings correlate with the ability of Spd2-DK to function as a PCM component to mediate microtubule nucleation during mitosis, despite its inability to recruit Fzr to the interphase centriole. By utilizing the Spd2-DK mutant protein, we addressed the specific function of the Spd2-Fzr interaction at the centrosome.

It was previously proposed that upon AO the centrosome-associated pool of Fzy first induces CycB destruction locally on the mitotic spindle. This then allows the dephosphorylation of centrosomal Fzr, which spreads into the cytoplasm to degrade the cytoplasmic fraction of CycB (ref. 16). Consistent with this model, we observed differential degradation kinetics between the centrosomal and cytoplasmic pools of CycB-GFP in *spd2*-null mutant NBs carrying the *RFP-spd2-WT* transgene (hereafter referred to as ‘Spd2-WT rescued NBs'); cytoplasmic CycB-GFP was destroyed at a slower rate than its centrosomal counterpart (Supplementary Fig. 11a,b, Supplementary Movie 5). This leads to a model that predicts that Fzr mislocalisation should affect the destruction kinetics of the cytoplasmic pool of CycB. However, *spd2*-null mutant NBs expressing RFP-Spd2-DK (hereafter called ‘Spd2-DK rescued NBs'), in which Fzr is mislocalized, showed comparable kinetics of cytoplasmic CycB-GFP destruction as Spd2-WT rescued NBs (Supplementary Fig. 11a,b, Supplementary Movie 6). This suggests that centrosomal Fzr localization is not rate-limiting for CycB degradation during mitotic exit. We found that the centrosomal localization of Fzy was unaffected by Spd2 depletion or the Spd2-DK mutation (Supplementary Fig. 12). Therefore, Fzy may play a dominant role in the spatiotemporal regulation of CycB degradation.

**Figure 6:**
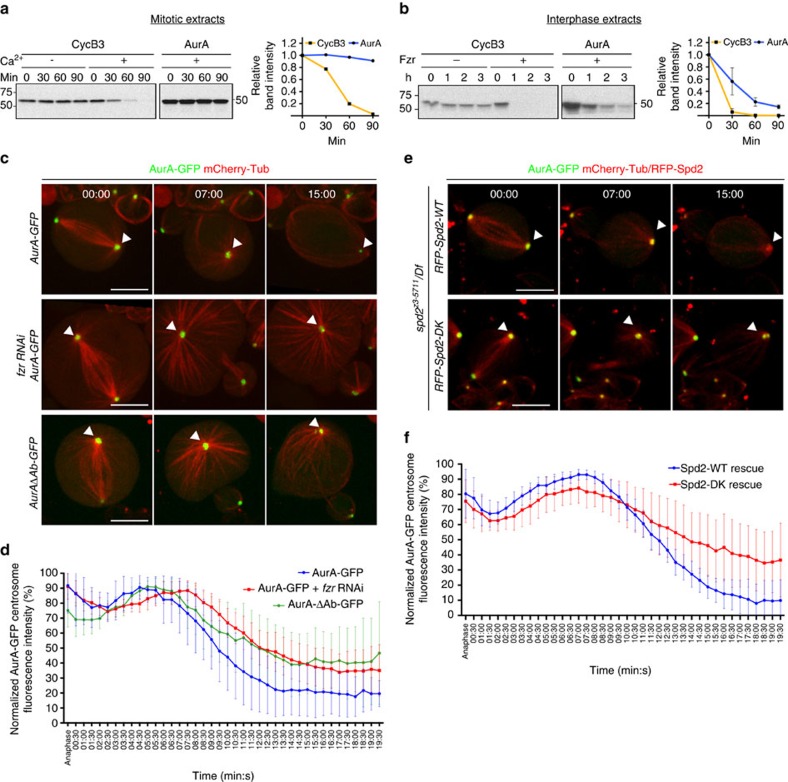
Centrosomal Fzr localization is required for timely Aurora A degradation during mitotic exit in the *Drosophila* NB. (**a**,**b**) Reconstituted APC/C-dependent destruction assays using ^35^S-labelled *Drosophila* AurA and Cyclin B3 (CycB3) as substrates. The band intensity in the autoradiographs was quantified and the mean values were plotted in line graphs (*n*=1 and 3, in mitotic and interphase extracts, respectively). Error bars in **b** indicate s.d. In mitotic frog egg extracts, CycB3, but not AurA, was degraded upon APC/C activation by addition of calcium (Ca^2+^, +, **a**), whilst both CycB3 and AurA were rapidly degraded upon addition of recombinant Fzr in interphase egg extracts (+Fzr, **b**). (**c**) Live imaging of NBs expressing GFP-fused wild type or the A-box mutant form of AurA, AurAΔAb (green), together with mCherry-Tubulin (red), induced with the NB-specific wor-Gal4 driver. The images of the NBs at selected time point are shown with the white arrowheads pointing to the apical centrosomes. (**d**) The centrosomal AurA-GFP signal intensity was quantified for each time point in the samples used in c, and the mean normalized value is presented in the line graph. Error bars, 95% CIs. In wild-type NBs, AurA-GFP started to decline at 5:30 min after anaphase and took around 8 min to reach its minimum value (blue line, *n*=8). AurAΔAb-GFP showed slower degradation kinetics (green line, *n*=12). *fzr* RNAi also significantly slowed down AurA-GFP degradation (red line, *n*=11). (**e**) Live imaging of the Spd2-WT or Spd2-DK rescued NBs expressing AurA-GFP (green) and mCherry-Tubulin (red). The images of the NBs at selected time point are shown with the white arrowheads pointing to the apical centrosomes. The Spd2-DK rescued NBs showed slower AurA-GFP degradation resulting in its higher retention 15:00 after AO, compared with the Spd2-WT rescued NBs. (**f**) The centrosomal signal intensity of AurA-GFP in the Spd2-WT or Spd2-DK rescued NBs was quantified and the mean normalized values are shown in the line graph. The Spd2-DK rescued NBs showed slower degradation kinetics of AurA-GFP and a higher residual signal of AurA-GFP (red line, *n*=12), compared to the Spd2-WT rescued NBs (blue line, *n*=13). Error bars 95% CIs. Scale bars, 5 μm.

The above results led us to examine Fzr-specific centrosomal targets. It was previously shown that vertebrate centrosomal Aurora A kinase (AurA) is targeted by APC/C^Fzr^, but not APC/C^Fzy^, for degradation via a unique degron motif A-box^39^,^40^,^41^. In reconstituted APC/C-dependent degradation assays^42^, we were able to observe rapid degradation of *Drosophila* AurA on the addition of Fzr in interphase *Xenopus* extracts, whilst it was completely stable in mitotic extracts containing Fzy (Fig. 6a,b). To confirm the involvement of APC/C^Fzr^ in AurA degradation *in vivo*, we monitored the kinetics of AurA-GFP during mitotic exit in live NBs. When induced by the NB-specific worniu-Gal4 driver (wor-Gal4), AurA-GFP was highly enriched at centrosomes during mitosis (Fig. 6c, Supplementary Movie 7). In control NBs, we found that centrosomal AurA-GFP fluorescence began to decrease ∼7:00 min after AO, then reached its minimum value (17.61% of maximum intensity) 18:00 min after AO (degradation rate, K^deg^: 7.372±0.7882% min^−1^, Fig. 6c,d Supplementary Fig. 13a), closely mirroring the kinetics of Fzr re-accumulation at centrosomes (Fig. 2e). Expression of *fzr* dsRNA significantly slowed AurA-GFP destruction (K^deg^: 5.160±0.4044% min^−1^, Supplementary Fig. 13a) with significantly higher residual fluorescence 19 min after AO (Fig. 6c,d Supplementary Movie 8). Although the A-box mutant of AurA (AurAΔAb) showed higher variability, a larger fraction of NBs (8 out of 12 samples) showed significant stabilization of centrosomal AurAΔAb-GFP signals (K^deg^: 6.349±0.44832% min^−1^, Fig. 6c, Supplementary Fig. 13a, Supplementary Movie 9). Thus Aurora A appears to be a Fzr substrate.

We then assessed the potential importance of the centrosomal localization of Fzr in APC/C activation during mitotic exit. The destruction kinetics of AurA-GFP in the Spd2-WT rescued NBs followed similar kinetics to control NBs; AurA-GFP intensity began to decrease 7:30 min after AO reaching the minimum at 18:00 min (K^deg^: 9.091±0.5485% min^−1^; Fig. 6e,f Supplementary Fig. 13b, Supplementary Movie 10). In contrast, the Spd2-DK rescued NBs showed significantly slower AurA-GFP destruction (K^deg^: 4.621±0.2560% min^−1^, Supplementary Fig. 13b) and substantially higher residual AurA-GFP signals (36.56%, Fig. 6e,f, Supplementary Movie 11). These results strongly suggest that Spd2-mediated recruitment of Fzr to the centriole is required for timely AurA destruction during mitotic exit.

**Figure 7:**
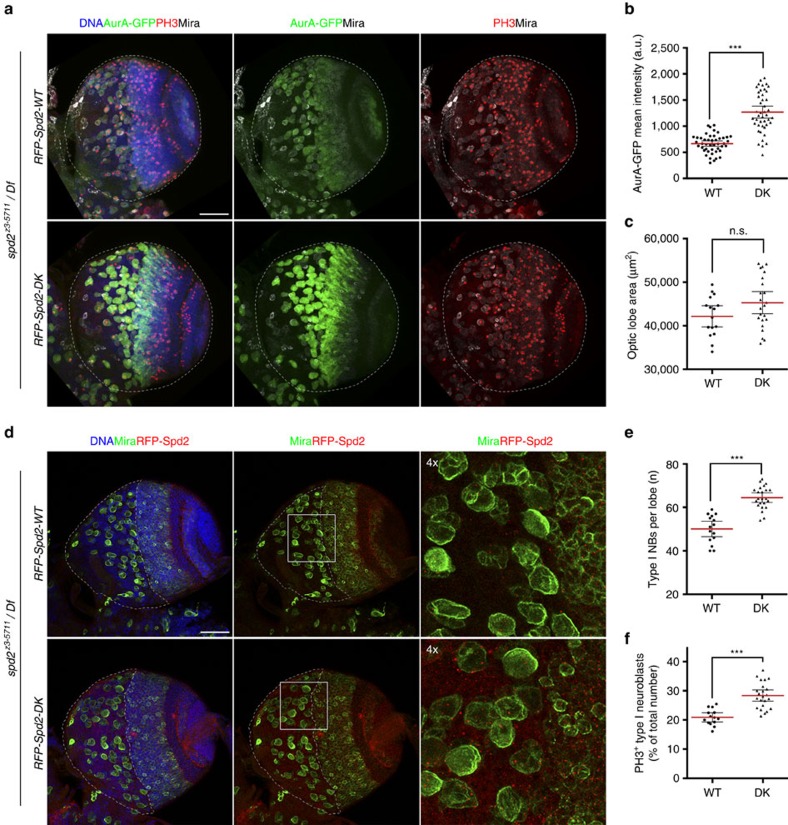
Centrosomal Fzr regulates proliferation and homeostasis of neural stem cells in the developing *Drosophila* brain. (**a**) Maximum intensity projections of the Spd2-WT or Spd2-DK rescued larval brains expressing AurA-GFP (green) induced with the wor-Gal4 driver, stained for DNA (blue), the mitotic marker phosphorylated histone H3 (PH3, red) and a NB marker Miranda (white). The Spd2-DK rescued brains accumulate AurA-GFP in NBs compared to the Spd2-WT rescue brains. The white dotted lines indicate the area used to measure the size of optic lobes. (**b**) The signal intensity of AurA-GFP was quantified in individual NBs (*n*=45) and presented in the dot plot. The red bars indicate the mean values and the error bars 95% CIs. The Spd2-DK rescued brains showed significantly more AurA-GFP than the Spd2-WT rescued brains (***P<0.0001, unpaired *t*-test). (**c**) Dot plot showing individual measurements of the optic lobe size in the Spd2-WT rescued and Spd2-DK rescued brains (*n*=16 and 23, respectively). The red bars indicate the mean values and the error bars 95% CIs. The optic lobe sizes were not significantly different between the two samples (n.s., not significant, *P*>0.05, unpaired *t*-test). (**d**) Maximum intensity projections of the Spd2-WT and Spd2-DK rescued larval brains stained for Miranda (green). The white dotted lines delimit the dorso-anterior parts of the optic lobes, which were used for the analysis of the NB number and the mitotic index of NBs. (**e**) Dot plot of the NB quantification for the Spd2-WT and Spd2-DK rescued brains (*n*=15 and 23, respectively). The red bars indicate the mean values and the error bars 95% CIs. The Spd2-DK rescued brains showed an increase in the number of type I NBs in the dorso-anterior lateral region (***P<0.0001, unpaired *t*-test). (**f**) Dot plot of the mitotic indexes in the Spd2-WT and Spd2-DK rescued brains (*n*=14 and 23, respectively). The red bars indicate the mean values and the error bars 95% CIs. The Spd2-DK rescued brains show a higher mitotic index than the Spd2-WT rescued brains (***P<0.0001, unpaired *t*-test). Scale bars, 50 μm.

*Drosophila* AurA has been shown to regulate proliferation and homeostasis of NBs in the developing central nervous system^43^,^44^. Thus we examined the effect of Fzr mislocalisation on the number and cell cycle progression of NBs in the Spd2-DK rescued larval brains. Consistent with the observed delay in AurA-GFP degradation (Fig. 6e,f), we detected significantly higher levels of AurA-GFP in NBs in the Spd2-DK rescued brains than in the Spd2-WT rescued brains (Fig. 7a,b). The size of the optic lobes in the Spd2-WT and Spd2-DK rescued brains was comparable (Fig. 7a,c). However, we observed a statistically significant increase in the number of type I NBs in the RFP-Spd2-DK brains in comparison to the Spd2-WT rescued brains (mean±95% confidence intervals (CIs): 64.48±1.074 versus 50.07±1.649, Fig. 7d,e). We also observed a subtle increase in mitotic cells in the NB population in the Spd2-DK rescued brains in comparison to the Spd2-WT rescued brains (mean±95% CIs: 28.37±0.9257% versus 20.89±0.7331%, Fig. 7a,f). These data highlight the potential importance of the spatial control of APC/C-dependent proteolysis in the regulation of cell cycle progression and stem cell homeostasis.

### Spd2 is an APC/C^Fzr^ substrate

**Figure 8:**
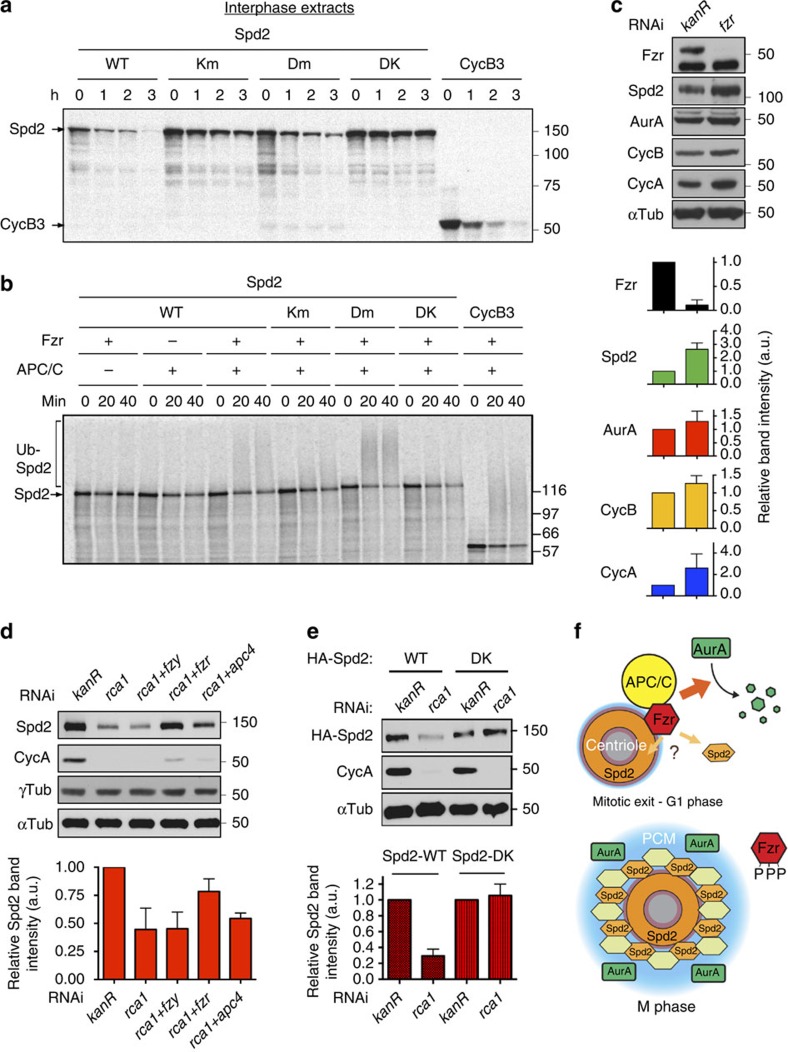
Spd2 is a novel APC/C^Fzr^ substrate. (**a**) APC/C-dependent destruction assays in interphase frog egg extracts using CycB3 and Spd2 as substrates. The control APC/C substrate CycB3 and wild type Spd2 (WT) were efficiently degraded upon Fzr addition in interphase egg extracts. The Km or Dm mutation partially stabilised Spd2, whilst the Spd2-DK mutant was not subject to APC/C-dependent degradation in interphase extracts. (**b**) Reconstituted APC/C-dependent ubiquitination assay using purified recombinant *Xenopus* APC/C. CycB3 and Spd2-WT, Dm, Km and DK were assayed for ubiquitination. Spd2-WT was polyubiquinated in the presence of both APC/C and Fzr, whilst Spd2-DK was hardly ubiquitinated. The signals corresponding to polyubiquinated and unmodified Spd2 are indicated by a bracket and an arrow. (**c**) Immunoblotting of the whole-cell extracts of *D.mel*-2 cells transfected with *kanR* or *fzr* dsRNA (top panels). Fzr depletion accumulates Spd2 and known APC/C substrates, CycA, CycB and AurA. The relative band intensities were quantified (bottom panels, *n*=4). Error bars, s.d. (**d**) Depletion of the APC/C^Fzr^-specific inhibitor Rca1 caused a significant reduction in the cellular levels of Spd2 and CycA, but not in γ-Tubulin, which were partially rescued by co-depletion of Fzr or an APC/C subunit Apc4, but not by Fzy co-depletion. The Spd2 band intensities were quantified and the mean values were presented in the bar graphs (bottom, *n*=3). Error bars, s.d. (**e**) HA-Spd2-WT, but not HA-Spd2-DK, was degraded upon Rca1 depletion in *D.mel-2* cells. CycA was used to monitor the efficiency of Rca1 depletion. The Spd2 band intensities in the immunoblots were quantified and the mean values were presented in the bar graphs (*n*=2). Error bars, s.d. (**f**) The model for the role of the centrosome recruitment of Fzr in APC/C-dependent proteolysis and the centrosome regulation. During mitotic exit, Fzr is recruited to the centrosome through the direct interaction with the centriolar pool of Spd2, where it targets AurA, as well as Spd2, for destruction, facilitating the conversion of the centrosome from its mitotic to interphase state. During mitosis, Fzr dissociates from the centriole, allowing AurA accumulation, whilst the PCM pool of Spd2 recruits other mitotic PCM components to nucleate microtubules.

Spd2 interacts with the WD40 repeat domain of Fzr via the D-boxes and the KEN-box in the same manner by which a canonical APC/C substrate is recognized by Fzr for APC/C-dependent degradation (Fig. 4). This raised the possibility that Spd2 may not only target Fzr at the centrosome but may also be targeted by the APC/C^Fzr^ for destruction. We directly tested this hypothesis by performing *in vitro* degradation assays using *Xenopus* egg extracts, where APC/C-dependent proteolysis can be recapitulated^42^. We found that Spd2 was efficiently degraded on addition of recombinant Fzr proteins in interphase egg extracts, but not in mitotic egg extracts containing endogenous Fzy (Fig. 8a, Supplementary Fig. 14a–d). We also performed *in vitro* ubiquitination assays using purified recombinant *Xenopus* APC/C (ref. 45) and found that Spd2 was efficiently polyubiquinated by the APC/C (Fig. 8b). Importantly, both Spd2 degradation and ubiquitination by APC/C^Fzr^ were dependent on the presence of the D-boxes and the KEN-box in Spd2; Spd2-DK was stable in interphase egg extracts and not ubiquitinated by APC/C^Fzr^ *in vitro* (Fig. 8a,b, Supplementary Fig. 14e).

To assess whether APC/C^Fzr^ regulates the cellular levels of Spd2 via proteolysis, we analysed the effect of either depleting or up-regulating the APC/C^Fzr^ on the cellular levels of Spd2 in *D. mel-2* cells. We found that *fzr* RNAi resulted in the accumulation of endogenous Spd2 as well as other known APC/C substrates such as CycB, Cyclin A and AurA (Fig. 8c). Consistently, Fzr depletion also led to a significant increase in the centrosomal Spd2 levels in interphase cells (Supplementary Fig. 14f), without affecting the ‘Zone II'-specific centriolar localization (Supplementary Fig. 14g,h). Conversely, depletion of the APC/C^Fzr^-specific inhibitor Rca1 (Emi1 or Xrp1 in human) gave rise to a dramatic reduction in endogenous Spd2 levels, but not in γ-Tubulin levels (Fig. 8d). This reduction in Spd2 levels on APC/C^Fzr^ upregulation is mediated by recognition of Spd2 by Fzr, as the cellular levels of HA-Spd2-DK were unaffected by Rca1 depletion (Fig. 8e). These results indicate that Spd2 is not only the centrosomal linker of Fzr but also a novel substrate of APC/C^Fzr^, implying the existence of a feedback loop between Fzr and Spd2.

## Discussion

In this study, we have addressed a long-standing hypothesis that APC/C-dependent proteolysis is spatially controlled by the subcellular localization of APC/C activators in metazoan cells. Specifically, we have determined that the APC/C activator Fzr localizes to the interphase centrioles through its interaction with Spd2 in *Drosophila*. We have shown that the centrosomal localization of Fzr is critical for APC/C-dependent destruction of AurA during mitotic exit and for NB homeostasis. To our knowledge, this is the first demonstration of the crucial role of a distinct cellular pool of APC/C activators in the spatial control of APC/C activity.

Our studies have uncovered two separate roles for the *bona fide* centrosome component Spd2 in *Drosophila*: an interphase role as the centrosomal linker for Fzr recruitment, and a mitotic role as a part of PCM recruited on centrosome maturation for microtubule nucleation. At present, we do not understand why Fzr is associated with the interphase centriolar pool of Spd2, but not with its mitotic PCM pool (Figs 1 and 2). We speculate, however, that modifications by mitotic kinases or the interaction of other PCM components with Spd2 may preclude the interaction between Fzr and Spd2. All known Spd2 orthologues share an essential function in centrosome maturation^33^,^34^,^46^,^47^. However, the *C. elegans* and vertebrate orthologues of Spd2 (SPD-2 and Cep192, respectively) are required for centriole duplication through ZYG-1/Plk4 recruitment, whereas *Drosophila* Spd2 is believed to be exclusively required for centrosome maturation and microtubule nucleation in mitosis^33^,^34^,^46^,^47^. It remains unclear whether the novel interphase function of *Drosophila* Spd2 to recruit the key cell cycle regulator Fzr is conserved in other Spd2 orthologues. We found that Cdh1 also localizes at the interphase centrosome in human cells, however, the human Spd2 orthologue Cep192 does not possess a KEN-box and did not interact with Cdh1 in our co-IP assays (Supplementary Fig. 5). An as-yet-unknown centrosomal component may therefore recruit Cdh1 to the centrosome in human cells.

Although we have established Spd2 as the centrosomal linker for Fzr (Fig. 5), molecular details of the physical interaction remain elusive. Spd2 directly binds Fzr's WD40 repeat domain via D-box and KEN-box motifs (Fig. 4), thereby potentially preventing Fzr from targeting APC/C substrates. However, our results instead point to a positive role of Spd2 for Aurora A degradation (Fig. 6). GFP-Fzr turns over rapidly at the centrosome^16^. Thus, Fzr might only transiently interact with Spd2. Alternatively, Fzr and Spd2 may cooperatively create a specific interface for the A-box, the APC/C degron unique to AurA (refs 40, 48). Future studies will be required to clarify the precise role of Spd2 in the APC/C targeting mechanism.

We demonstrated that Spd2 is also an APC/C^Fzr^ substrate (Fig. 8). It is known that some APC/C regulators, including UbcH10/Vihar and fission yeast Mes1, are also targeted by the APC/C for destruction, creating feedback loops^49^,^50^. By forming a negative feedback loop, Spd2 may maintain the optimal level of the Fzr pool at the interphase centrosome (Fig. 8f). It remains to be determined how such a feedback loop may affect the centrosome or APC/C activity, and how Spd2 proteolysis coordinates with its role in the centrosomal recruitment of Fzr.

The APC/C targets numerous key centrosomal regulators, including AurA, Plk1 and Nek2, whose over-expression is prevalent in cancer cells^51^,^52^. Tethering Fzr to the centrioles may allow the APC/C to efficiently target its centrosomal substrates to couple the centrosome function to cell cycle progression. We showed that developing *Drosophila* larval brains in which Fzr is not present at the centrosomes exhibit the accumulation of AurA in NBs, concomitantly with an increased population of mitotic NBs (Fig. 7). These findings point to the potential importance of centrosome-associated APC/C activity in organ development and stem cell homeostasis. It is noteworthy that the APC/C-specific E2 UbcH10 and its inhibitor Emi1 are also enriched at the centrosomes^18^,^53^,^54^. The future identification of the centrosomal receptors for each APC/C regulator will assist in further elucidating the roles of the APC/C in centrosome regulation.

## Methods

### DNA constructs

cDNA clones for *spd2, aurA, fzr, fzy* and *cdc27* were obtained from the *Drosophila* Genomics Resource Center (DGRC). Entry clones with the coding sequences encoding full length or fragments of these genes were generated using Gateway System (Thermo Fisher Scientific). Expression constructs were made by recombination between entry clones and the following destination vectors: pDEST15 (for N-terminal GST fusion in *E. coli*, Thermo Fisher Scientific), pAGW (for actin5C promoter-driven N-terminal GFP fusion in *D. mel-2* cells, DGRC), pMT-N-GFP, FLAG or HA (for inducible metallothionein promoter-driven N-terminal GFP, 3xFLAG or 3xHA fusion in *D. mel-2* cells), pMTB-N-ProA (ref. 55; for female germ-line specific expression of N-terminal Protein A fusion in flies), pURW (for ubiquitin promoter-driven N-terminal RFP fusion in flies) and pPWG (for gal4-driven expression of C-terminal GFP fusion).

The *spd2-Dm, spd2-Km* and *spd2-DK* mutant genes were generated by QuickChange Site-Directed Mutagenesis Kit (Agilent Technologies) using the spd2 entry clone as a template. To generate *spd2-Dm* and *spd2-Km*, the first and forth amino acids in the four conserved D-box consensus sequences (RxxL, located at positions: 480–483, 620–623, 636–639 and 667–670) and all amino acids in the KEN-box consensus (KEN, at 436–438) in the Fzr binding region were mutated to Alanine, respectively. *spd2-Dm* also possesses mutations: R426A and L428A. *spd2-DK* was created by mutating the KEN-box sequence in the *spd2-Dm* gene. The *aurAΔAb* mutant gene was generated by deleting the DNA sequence corresponding to the 46–85th amino acids from the wild-type *aurA* sequence.

The series of truncated constructs of *spd2* were generated by amplifying each segment using PCR and then cloning them into pDONR221 vector using Gateway System. The genes were then transferred into either pDEST15 (for expression of GST fusions in *E. coli*) or pAGW (for expression of N-terminal GFP fusions in *D. mel-2* cells) by recombination. His-Fzr constructs used for *in vitro* transcription and translation were generated by inserting the full length, or N160 or C318 segments of the *fzr* coding sequence, into the pHY22 vector linearized with NcoI and BamHI restriction enzymes. The DNA sequences of all the constructs generated were confirmed by Sanger DNA sequencing (Source Bioscience).

### *Drosophila* strains

All crosses were raised at 25 °C under standard conditions. The following stocks (described in FlyBase, unless otherwise stated) were used: *Oregon R* (as the wild-type control), *spd2*^*Z3*−*5711*^ (ref. 33)*, Df(3R)BSC561*, *fzr*^*ie8f*^ (ref. 27), *pUbq-GFP-fzr*, *pUbq-GFP-fzy* (ref. 16), *wor-gal4* (ref. 56), *UAS-mCherry-tubulin* (ref. 57), *tubulin-GFP* (ref. 58), *pUbq-CycB-GFP* (ref. 59) and *UAS-fzr RNAi* (v25550, VDRC). The fzr-GFP^fosmid^ line was generated in the *Drosophila* TransgenOme Project^26^. This transgenic fly line carries the fosmid DNA containing ∼36 kb-long genomic sequences of the *Drosophila* X-chromosome including the entire *fzr* gene locus. The *fzr* gene is fused to a 2xTY1-GFP-V5 tag at its 3′ terminus and is expressed under the control of its native regulatory elements.

For the generation of the transgenic pUbq-RFP-spd2-WT and pUbq-RFP-spd2-WT fly lines, the *spd2-WT* or *spd2-DK* genes were transferred from the entry clones to pURW destination vector by recombination. For the generation of the UASp-aurA-GFP and UASp-aurAΔAb-GFP transgenic fly lines, the wild-type *aurA* or *aurAΔAb* genes were transferred to a pPGW vector by recombination. The resulting plasmids were injected into *w*^*1118*^ embryos using the microinjection service of the Fly Facility at the Department of Genetics, University of Cambridge.

### Antibodies

The following antibodies were used for western blotting (WB) and immunofluorescence (IF): rabbit anti-Spd2 (1:1,000 for WB and IF (ref. 60)), rabbit anti-Fzr, rabbit anti-Fzy and rabbit anti-Cdc27 (gifts from Jordan Raff, 1:1,000 for WB (refs 16, 23)), mouse anti-GFP (Sigma-Aldrich, 11814460001, 1:1,000 for WB), mouse anti-HA (Covance HA11, 1:1,000 for WB and IF), mouse anti-FLAG (M2, Sigma, F3165, 1:1,000 for WB and IF), mouse anti-α-Tubulin (DM1A, Sigma-Aldrich, 1:5,000 for WB, 1:2,000 for IF), mouse anti-PSTAIRE (Sigma P7962, 1:4,000 for WB), rabbit anti-Cnn (ref. 61; 1:1,000 for WB and IF), mouse anti-γ-Tubulin (GTU-88, Sigma T6557, 1:200 for IF), chicken anti-Dplp (ref. 62; 1:1,000 for IF), rabbit anti-Asl (ref. 63; 1:2,000 for IF), guinea pig anti-Asl (a gift from Nasser Rusan^64^, 1:40,000 for IF).

### Cell culture and DNA and dsRNA transfection

*Drosophila D.mel-2* cells (Thermo Fisher Scientific) were cultured in Express Five SFM medium (Thermo Fisher Scientific) supplemented with 2 mM L-glutamine and Pen Strep (Thermo Fisher Scientific). DNA transfection was performed using FuGene HD transfection reagent (Promega). DNA (3 μg) were mixed with 15 μl of transfection reagent in 150 μl of nuclease-free water and incubated 15 min at room temperature. The mixture was then added to 2 × 10^6^ cells previously seeded on a well of a six-well plate in a final volume of 2 ml. Stable cell lines were generated by adding the antibiotic 48 h after transfection. RNAi experiments were performed using TransFast transfection reagent (Promega). dsRNA (30 μg) were mixed with 20 μl of transfection reagent in 1 ml of medium and incubated 15 min at room temperature, then added to 2 × 10^6^ cells. Four and two rounds of dsRNA transfection were performed to efficiently deplete endogenous Spd2 and Fzr, respectively.

Oligonucleotide primers used to generate dsRNA are listed below: kanR-F: 5′- TAATACGACTCACTATAGGGAGAGACAATCTATCGCTTGTATG -3′

kanR-R: 5′- TAATACGACTCACTATAGGGAGAGGAATCGAATGCAACCGGCGC -3′

spd2exon-F: 5′- TAATACGACTCACTATAGGGAGAGTCGCGTTCCAGCCAAGCAAAGA -3′

spd2exon-R: 5′- TAATACGACTCACTATAGGGAGATCCCCCACCTCCGTTAAGACTCAG -3′

spd2UTR-F1: 5′- TTTGATCGAAGCGACGCGCCTTTTTTTTGTTTTCGCGTTCGCA -3′

5′- CTGCAAACTGTAACTGTTTAATTACAAGCGGAAATTTGTTTTATTTGTGCCTG -3′

spd2UTR-R1: 5′- TAATACGACTCACTATAGGGAGATATACTTTATTAGTTTTTA -3′

spd2UTR-F2: 5′- AATACGACTCACTATAGGGAGATTTGATCGAAGCGACGCGCCT -3′

For generation of dsRNA against spd2UTR, first, the 3′UTR sequence of the *spd2* cDNA clone was amplified using spd2UTR-F1 and spd2UTR-R1 primers. The resulting DNA fragment was then used to amplify the 5′UTR sequence of the *spd2* cDNA together with spd2UTR-F2 primer.

### Identification of Fzr interactors

For purification of PrA-Fzr from *D. mel-2* cells (Fig. 3a,b), 10 × 10^9^ cells expressing PrA-Fzr were lysed in 10 ml lysis buffer (LB; 50 mM K-HEPES pH 7.5, 100 mM AcOK, 100 mM NaCl, 50 mM KCl, 2 mM MgCl2, 0.1% NP40, 2 mM EGTA-Na, 5% glycerol, 1 mM dithiothreitol (DTT), PhosSTOP phosphatase inhibitor and complete protease inhibitor cocktail) using Power Gen 125 homogenizer (Thermo Fisher Scientific)^65^. For purification from *Drosophila* embryos, 2 g of embryos expressing PrA-Fzr under the *maternal α-tubulin* promoter were homogenized in 10 ml LB using Dounce tissue homogenizer (Wheaton)^55^. The extracts were centrifuged at 10,000*g* at 4 °C for 10 min, and the clarified lysates were applied to pre-washed rabbit immunoglobulin-G-conjugated Dynabeads M-450 Epoxy (Thermo Fisher Scientific) at 4 °C for 2 h. The beads were washed three times in LB and bound proteins were eluted with three rounds of elution with 1 M NH_4_OH. The eluted proteins were then precipitated in acetone and analysed by liquid chromatography coupled to tandem MS using Nano-Acquity (Waters) LC system and Orbitrap Velos mass spectrometer (Thermo Electron Corp., San Jose, CA, USA) at the Mass Spectrometry Laboratory, Institute of Biochemistry and Biophysics, Polish Academy of Sciences (Warsaw, Poland). Protein samples were subjected to a standard ‘in-solution digestion' procedure, in which proteins were reduced with 100 mM DTT, alkylated with 0.5 M iodoacetamide and digested overnight with trypsin (Promega). The peptide mixture was applied to an RP-18 precolumn (Waters) using 0.1% trifuloroacetic acid (TFA) as the mobile phase and then transferred to a nano-HPLC RP-18 column (Waters) using a 0–60% acetonitrile gradient for 120 min in the presence of 0.05% formic acid with the flow rate of 150 nl min^−1^. Column outlet was directly coupled to the ion source of the spectrometer operating in the regime of the data-dependent MS to MS/MS switch. A blank run preceded each analysis to ensure lack of cross contamination from any previous samples. Acquired raw data were processed using Mascot Distiller followed by Mascot Search (Matrix Science, London) against the FlyBase database. Search parameters for precursor and product ion mass tolerance were 20 p.p.m. and 0.6 Da, respectively, with search parameters set as follows: one missed semi-Trypsin cleavage site allowed, fixed modification of cysteine by carbamidomethylation and variable modification of lysine carbamidomethylation and methionine oxidation. Peptides with Mascot scores exceeding the threshold value corresponding to the <5% false positive rate were considered to be positively identified.

The proteins identified in the PrA-Fzr samples were compared with those identified using various negative controls (ProA tag only, no bait and so on) or other bait proteins, including Fzy and APC/C subunit Cdc27, by utilizing the DAPPER database^66^, and potential Fzr-specific interactors were listed for further analysis. Amongst these, Spd2 was one of the proteins that showed high Mascot scores (>100).

### Co-immunoprecipitation

For co-IP (Fig. 3g, Supplementary Fig. 5d), antibody-coupled magnetic beads were prepared by immobilizing the antibodies to PrA-coupled Dynabeads (Thermo Fisher Scientific) according to the manufacturer's instructions. *D. mel-2* cell extracts were prepared by harvesting ∼2 × 10^7^ cells and lysing in 500 μl of buffer containing 50 mM K-HEPES pH 7.5, 100 mM KOAc, 100 mM NaCl, 50 mM KCl, 2 mM MgCl_2_, 0.1% NP40, 1 mM DTT, 2 mM EGTA, 5% glycerol, 50 nM Okadaic acid, 1 mM PMSF, EDTA-free complete protease inhibitor cocktail and PhosStop (Roche). The extracts were incubated with the beads for 2 h at 4 °C. For immunoprecipitation of GFP-tagged proteins (Figs 3c–f and 4b,c,g,h, Supplementary Fig. 5e), *D. mel-2* cell extracts were prepared by incubating ∼2 × 10^7^ cells in 200 μl of Lysis buffer containing 10 mM Tris-HCl pH 7.5, 150 mM NaCl, 0.5 mM EDTA, 0.5% NP40, 50 nM Okadaic acid, 1 mM PMSF, EDTA-free complete protease inhibitor cocktail and PhosStop, on ice for 30 min. Lysates were clarified by centrifugation (4 °C, 10,000 r.p.m., 10 min) and diluted to a final volume of 500 μl in lysis buffer without NP40. The resulting lysates were then incubated with 10 μl GFP-Trap-A beads (ChromoTek) for 2 h at 4 °C. For immunoprecipitation of Fzr-GFP with larval brain extracts (Supplementary Fig. 1c), 30 brains were dissected from third instar larvae in PBS containing complete protease inhibitor cocktail (Roche), then resuspended in 150 μl of lysis Buffer and homogenized using pestles. The lysate were clarified by centrifugation (4 °C, 10,000 r.p.m., 10 min) and applied to 15 μl of GFP-Trap-MA beads (ChromoTek). After washes, immunoprecipitated proteins were eluted by boiling the beads in Laemmli buffer and were analysed by WB. Whole western blot membranes used in this study are shown in Supplementary Fig. 15.

For quantification of the co-IP efficiency (Figs 3d,f and 4c,h), signal intensities of the bands corresponding to the bait and the prey in the blots were measured by using the Gel Analyser tool in ImageJ. The precipitation efficiency of the prey (Fig. 3f) was defined as the ratio of the value of its IP fraction to the value of its input fraction (normalized by its dilution). The co-IP efficiency of a bait protein with a specific prey protein (Figs 3d and 4c,h) was then determined by dividing the precipitation efficiency of the prey protein by the value of the IP fraction of the bait for normalization.

### *In vitro* binding assays

The bait GST and GST-Spd2 fusion proteins were expressed in *E. coli* BL21-CodonPlus cells (Agilent) and were purified using Glutathione Sepharose 4B resin (GE Healthcare) according to the manufacturer's instructions. ^35^S-methionine-labelled proteins were prepared using the TnT T7 Quick Coupled Transcription/Translation System (Promega), according to the manufacturer's instructions. Binding assays were performed by mixing a bait protein on the beads with the ^35^S-labelled proteins in 300 μl binding buffer containing 50 mM HEPES pH 7.5, 1 mM EGTA, 1 mM MgCl_2_, 0.1% Triton X-100, 150 mM NaCl, 0.5 mg ml^−1^ BSA, 1 mM DTT and EDTA-free complete protease inhibitor cocktail (Roche). Suspensions were incubated with gentle agitation for 30 min at room temperature. The beads were then washed several times in the binding buffer before boiling in 25 μl Laemmli buffer. Samples were resolved by SDS–PAGE and stained with Coomassie Blue. The resulting gels were dried and used for autoradiography (Fig. 4d,i,j, Supplementary Figs 6 and 7).

For quantification of the pull-down efficiency (Fig. 4j, Supplementary Figs 6b and 7b), signal intensities of the bands corresponding to the prey proteins on the autoradiography were measured using the Gel Analyser tool in ImageJ. The pull-down efficiency of each GST fusion with a prey protein was determined by dividing the value of the precipitated fraction of the prey by the value of its input fraction, taking into account the dilution of the input.

### Immunocytochemistry of *D. mel-2* cultured cells

*D. mel-2* cells were plated on non-treated or 0.5 mg ml^−1^ concanavalin-A-coated coverslips. For detection of endogenous Fzr (Figs 1d and 2a), *D. mel-2* cells were pre-extracted by dipping into a BRB-80 solution (80 mM K-Pipes pH 6.8, 1 mM MgCl_2_, 1 mM EGTA pH 6.8) containing 0.1% NP40 for 6–8 s. The cells were then fixed for 30 min with 4% formaldehyde in BRB-80, before being incubated for 10 min in BRB-80 containing 0.1% Triton X-100. In all the other cases, cells were fixed in ice-cold methanol for 10 min at room temperature, washed three times in PBS, then blocked in PBSTB (3% BSA, 0.5% Triton X-100 in PBS) for 30 min at room temperature. Coverslips were then incubated with primary antibody solution for 1 h at room temperature, washed three times in PBSTB and incubated with secondary antibody solution for 2 h at room temperature. Coverslips were washed three times in PBS, rinsed in pure water and mounted on slides using Vectashield anti-fade mounting medium (Vectorlab). Microscopic analysis was performed on Axiovert 200M microscope (Carl Zeiss) or Nikon Ti-E inverted microscope. Images were acquired with a Cool SNAP HQ2 camera (Photometrics) using Metamorph (MDS Analytical Technologies), or with DS-Qi1Mc camera using NIS-Elements software (Nikon). The resulting data were analysed using ImageJ (National Institutes of Health) and processed in Photoshop (Adobe).

### Structured illumination microscopy

For 3D-SIM microscopy (Fig. 1f,g, Supplementary Figs 4b,c; 8c,d and 14g,h), *D. mel-2* cells were seeded on Concanavalin-A-coated No. 1.5H precision coverslips and allowed to settle for 1 h, before fixation and permeabilization in ice-cold methanol for 10 min. The rest of the staining procedure was performed as outlined above. Super resolution microscopy was performed on a DeltaVision OMX V3 (Applied Precision) equipped with a × 100 1.4 NA oil objective (Olympus)^30^. Reconstruction and alignment of the 3D-SIM images was performed using softWoRx (Applied Precision). Maximum intensity projections were generated with ImageJ (National Institutes of Health) and panels assembled in Adobe Photoshop.

### Immunostaining of *Drosophila* tissues

For fixation of *Drosophila* embryos (Fig. 1a,b), embryos were collected and fixed with 8% formaldehyde in PBS with the same volume of Heptane in a glass vial for 20 min at room temperature. After vigorous shaking, embryos in the formaldehyde layer were collected and further fixed with ice-cold methanol. After re-hydration with PBS, embryos were pre-incubated with PBSTB (PBS containing 0.05% Triton X-100, 1% BSA) for at least 15 min at room temperature.

For fixation of larval NBs (Figs 1c, 2c and 5c–f, 7, Supplementary Figs 9 and 12d,e), larval brains were dissected from third instar larvae in PBS and then transferred to a solution of 4% formaldehyde in PBS supplemented with MgCl_2_ and EGTA for 25 min. The brains were then washed with PBS-Triton 0.3% and pre-incubated with PBSTB (PBS containing 0.3% Triton, 3% BSA).

For fixation of ovaries (Fig. 2b, Supplementary Fig. 2), ovaries were dissected in PBS supplemented with 0.2% Tween 20 (PBT) from well-fed adult female flies. Ovaries were fixed in 4% paraformaldehyde in PBT for 20 min. Following the wash with PBT, the ovaries were pre-incubated in PBSTB (PBS containing 0.2% Tween 20, 10% BSA) for 1 h.

The fixed tissue were then incubated with the primary antibodies in PBSTB overnight at 4 °C. After three washes in PBSTB, samples were incubated in PBSTB with the secondary antibodies (1:1,000) and DAPI (1:1,000) for 2 h at room temperature. After three washes in PBSTB, tissue samples were mounted in Vectashield. Samples were analysed on the Nikon C2 confocal microscope. The images were processed using the NIS-Elements software or ImageJ.

### Live imaging of *Drosophila* larval NBs

For live imaging of primary culture of larval NBs (Figs 2d,e and 6, Supplementary Figs 4f,g and 11), third instar larvae were dissected in PBS and slightly squashed to improve resolution of the centrosome signals. Images were acquired on a Zeiss Axiovert200 microscope fitted with a PerkinElmer RSIII spinning disk confocal unit (PerkinElmer Life Sciences) and running the Volocity v6.3. Single optical sections were captured at 30-sec intervals (unless otherwise stated) with a × 100 lens (N.A. 1.4). Data sets were imported into ImageJ and Photoshop for movie export and figure generation, respectively.

### Centrosomal signal intensity measurement

All the signal intensity measurements were carried out using ImageJ or NIS-element. For the measurement of centrosomal signal intensities in *D. mel-2* cells (Supplementary Figs 4e, 5b, 12b and 14f), a small circle was drawn around a centrosome (as indicated by the reference centrosome marker) on a maximum intensity projection, and the mean value in the appropriate channel was measured and used as Sample (S). Three additional points outside of the centrosome were measured: two in close proximity to the centrosome and one in the empty background of the slide. The average of these three measurements was used as Background (B). To get the corrected centrosome intensity measurement the following equation was applied: S^correct^=(S−B) × B^−1^.

For the measurement of centrosomal signal intensities in NBs using fixed larval brains (Fig. 5e,f, Supplementary Figs 9b–d and 12e), confocal images of the dorso-anterior part of optic lobes were taken and maximum intensity projections of individual NBs were generated. Then, in the projection images, two equal-sized regions of interest (ROI) were selected for each centrosome: one containing the centrosome and one in its proximity. The mean values were measured in each of the regions and were used as Sample (S) and Background (B), respectively. The centrosomal signal intensity (S^cen^) was defined as S−B. In the interphase NB, the apical centrosome and the basal centrosome were determined based on RFP-Spd2 values: the apical centrosome has a higher RFP-Spd2 value than the other.

For the measurement of centrosomal signal intensities using the time-lapse NB images (Figs 2e and 6d–f, Supplementary Figs 4g and 11b), S^cen^ for the apical centrosome (one segregated into a daughter NB after division) was determined at each time point over the time course as described above. The values were then normalized using the highest S^cen^ in each time course as the reference. For the measurement of the cytoplasmic signal intensities of CycB-GFP (Supplementary Fig. 11b), two ROI were selected for each cell: one ROI in the cytoplasm and the other ROI in the vicinity outside the cell. The mean values in each of the regions were used as S and B, respectively, and the cytoplasmic signal intensity (S^cyto^) was defined as S-B.

To determine the K^deg^ of AurA-GFP (Supplementary Fig. 13), the mean values of AurA-GFP fluorescence between the time of the onset of AurA-GFP degradation and the time when the AurA-GFP signal reached the minimum in control NBs (7:00 min and 14:00 min after AO, respectively) were plotted in a line graph and the linear regression was used to determine the best-fit line in GraphPad Prism. The slope of the best-fit line corresponds to the negative value of the K^deg^ of the reaction.

### AurA-GFP signal measurement in whole-mount larval brains

For the AurA-GFP signal measurements, confocal images of the dorso-anterior parts of the optic lobes, where only Type I NBs are present^67^, were taken by the Nikon C2 confocal microscope. NB areas were selected using the Polygon selection tool in ImageJ on the maximum intensity projections of the brains by using Mira as reference and the mean grey values of the AurA-GFP signal were quantified.

### Optic lobe size measurement in whole-mount larval brains

Optic lobe areas were measured in the maximum intensity projections of the larval brains using the Polygon selection tool of ImageJ 1.50i software (NIH, Bethesda, MA, USA), considering the limits of the dorsal brain optic lobe, and represented as μm^2^ (Fig. 7c).

### Measurement of NB number and mitotic index in brains

To determine the number of Type I NBs (Fig. 7e), we focused our analysis on the dorso-anterior region of the third instar larval brain, which contains only Type I NBs, but not more proliferative Type II NB (ref. 67). NBs were identified based on their large size and Miranda staining. The NBs in each optic lobe were counted by using the cell counter plugin in ImageJ. The mitotic index (the ratio of the number of mitotic NBs to the total NB number) was determined by using the phospho-histone H3 (PH3) signal as a marker of mitotic NBs (Fig. 7f).

### *In vitro* APC/C-dependent destruction and ubiquitination

The *in vitro* destruction assays were performed in *Xenopus* egg extracts using ^35^S-methionine-labelled substrate proteins prepared in a coupled *in vitro* transcription–translation system (Promega; Fig. 8a, Supplementary Fig. 14a–e). For the mitotic destruction assay, labelled substrates were incubated in cytoplasmic extracts of cytostatic factor-arrested *Xenopus* eggs. The reactions were then started by adding 0.4 mM CaCl_2_ and 10 μg ml^−1^ cycloheximide followed by incubation at 23 °C with or without the purified APC/C inhibitor Mes1. Aliquots were collected into 2 × Laemmli buffer at 0, 30, 60 and 90 min and resolved by SDS–polyacrylamide gel electrophoresis (SDS–PAGE). The gel was dried and used for autoradiography. For interphase destruction assays, the cytostatic factor extracts were first released into interphase by addition of 0.4 mM CaCl_2_ and 10 μg ml^−1^ cycloheximide followed by the incubation of 2-4 h at 23 °C. Substrates were added to the extracts and the reactions were then started by adding purified *Xenopus* Fzr to the mixture. Aliquots were collected at 0, 1, 2 and 3 h.

The *in vitro* ubiquitination assays (Fig. 8b) were performed using purified *Xenopus* APC/C with ^35^S-methionine-labelled substrate proteins. Recombinant *Xenopus* APC/C containing Apc6 subunit fused to tobacco etch virus (TEV) protease cleavable tandem Strep II-tag was expressed in High Five insect cells (Thermo Fisher Scientific) and purified using Strep-Tactin Superflow Column (Qiagen)^45^. Ubiquitination reactions were performed at 23 °C in 20 μl of buffer (20 mM Tris-HCl, pH 7.5, 100 mM KCl, 2.5 mM MgCl2, 2 mM ATP, 0.3 mM DTT) containing 0.075 mg ml^−1^ purified recombinant *Xenopus* APC/C, 0.05 mg ml^−1^ E1, 0.025 mg ml^−1^ UbcX, 0.75 mg ml^−1^ ubiquitin, 1 μM ubiquitin-aldehyde, 150 μM MG132, 280 nM purified His-Cdh1 protein and 1 μl of ^35^S-labelled substrates. The reactions were stopped at the indicated time points with SDS sample buffer and resolved by SDS–PAGE followed by autoradiography.

### Statistical analyses

Statistical analysis was performed with GraphPad Prism. D'Agostino and Pearson omnibus normality test was applied to data sets to assess data distribution. For normally distributed data, unpaired *t*-test was used. For non-normally distributed data, Mann–Whitney *U* test was used. Differences are considered significant with a *P* value <0.05. * denotes 0.01<*P*≤0.05, **0.001<*P*≤0.01, ***0.0001≤*P*≤0.001 and ****P≤0.0001.

### Data availability

The data that support the findings of this study are available from the corresponding author upon request.

## Additional information

**How to cite this article:** Meghini, F. *et al*. Targeting of Fzr/Cdh1 for timely activation of the APC/C at the centrosome during mitotic exit. *Nat. Commun.* 7:12607 doi: 10.1038/ncomms12607 (2016).

## Supplementary Material

Supplementary Information Supplementary Figures 1-15

Supplementary Movie 1 GFP-Fzr dynamics in mitotic neuroblasts. mCherry-Tubulin (red) was used to monitor mitotic progression. GFP-Fzr (green) dissociates from the centrosomes during mitosis.

Supplementary Movie 2 GFP-Fzy dynamics in mitotic neuroblasts. mCherry-Tubulin (red) was used to monitor mitotic progression. GFP-Fzy (green) dissociates from the centrosomes and accumulates at the kinetochores after nuclear envelope breakdown.

Supplementary Movie 3 Mitotic progression in *spd2*-null mutant neuroblasts expressing RFP-Spd2- WT (red). Tubulin-GFP (green) was used to monitor mitotic progression. The Spd2- WT rescued NBs undergo mitosis normally.

Supplementary Movie 4 Mitotic progression in *spd2*-null mutant neuroblasts expressing RFP-Spd2- DK (red). Tubulin-GFP (green) was used to monitor mitotic progression. The Spd2- DK rescued NBs undergo mitosis normally.

Supplementary Movie 5 CycB-GFP degradation in *spd2*-null mutant neuroblasts expressing RFPSpd2-WT. CycB-GFP (in green) shows the biphasic degradation pattern during mitotic exit: it starts to disappear from the centrosomes and the spindle at a fast rate, and then later degrades in the cytoplasm at a slower rate.

Supplementary Movie 6 CycB-GFP degradation in *spd2*-null mutant neuroblasts expressing RFPSpd2-DK. CycB-GFP (green) shows the biphasic degradation pattern during mitotic exit in the Spd2-DK recued NBs, indistinguishable from the Spd2-WT recued NBs.

Supplementary Movie 7 AurA-GFP degradation in control neuroblasts. mCherry-Tubulin (red) and AurA-GFP (green) were induced by wor-gal4 driver in NBs. mCherry-Tubulin was used to monitor mitotic progression. AurA-GFP begins to disappear from the centrosomes during late anaphase.

Supplementary Movie 8 AurA-GFP degradation in fzr RNAi NBs. mCherry-Tubulin (red), AurAGFP (green) and fzr dsRNA were induced by wor-gal4 driver in NBs. mCherryTubulin was used to monitor mitotic progression. The degradation of AurA-GFP is slower in fzr RNAi NBs, compared to control NBs.

Supplementary Movie 9 AurAΔAb-GFP degradation. mCherry-Tubulin (red) and AurAΔAb -GFP (green) were induced by wor-gal4 driver in NBs. AurAΔAb-GFP is degraded much slower than AurA-GFP.

Supplementary Movie 10 The degradation of AurA-GFP in *spd2*-null mutant neuroblasts expressing RFP-Spd2-WT (Spd2-WT rescued NBs). mCherry-Tubulin (red) and AurA-GFP (green) were induced by wor-gal4 driver. mCherry-Tubulin was used to monitor mitotic progression. AurA-GFP degradation in the Spd2-WT rescued NBs is comparable to that in control NBs.

Supplementary Movie 11 AurA-GFP degradation in *spd2*-null mutant neuroblasts expressing RFP-Spd2-DK (Spd2-DK rescued NBs). mCherry-Tubulin (red) and AurA-GFP (green) were induced by wor-gal4 driver. mCherry-Tubulin was used to monitor mitotic progression. AurA-GFP (green) degradation in the Spd2-DK rescued NBs is much slower than in the Spd2-WT rescued NBs.
