# Supplementary material for: Targeting of Fzr/Cdh1 for timely activation of the APC/C at the centrosome during mitotic exit
Source: Nat Commun. 2016 Aug 25;7:12607. doi: 10.1038/ncomms12607 (PMC5007356; doi:10.1038/ncomms12607)
Supplement: Supplementary Information — Supplementary Figures 1-15 [file ncomms12607-s1.pdf]

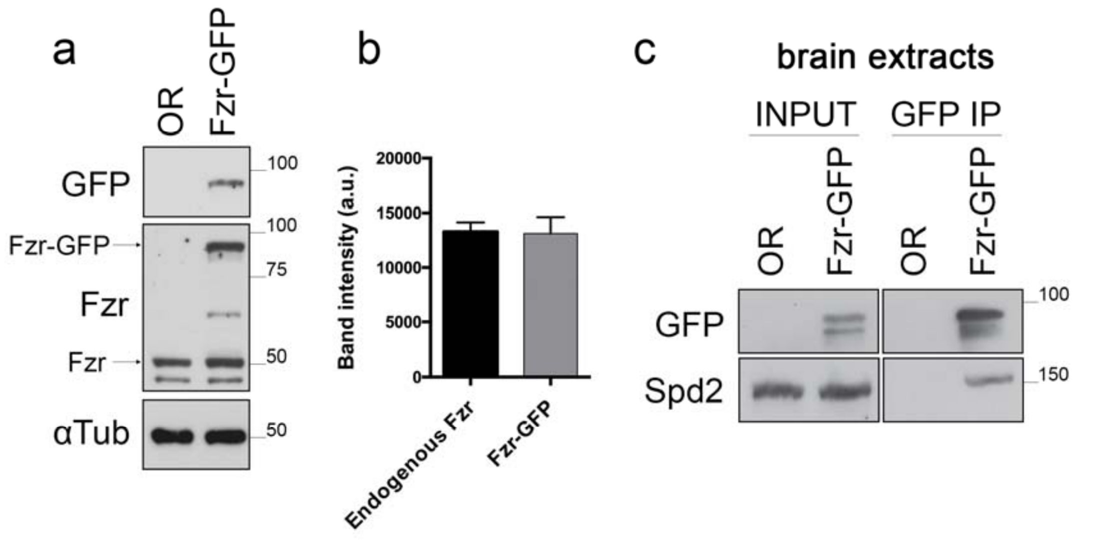

Supplementary figure 1. Fzr-GFP^{fosmid} is expressed at the comparable level to endogenous Fzr and interacts with Spd2 in *Drosophila* brain tissues

a) Immunoblotting of the brain extracts from the third instar larvae of wild type (Oregon R, OR) and the *fzr-GFP^{fosmid}* line. Fzr-GFP^{fosmid} was detected with anti-GFP and anti-Fzr antibodies. **b)** Quantification of the signal intensity of the bands corresponding to endogenous Fzr and Fzr-GFP^{fosmid} in the anti-Fzr immunoblotting in a. The mean values are shown in the bar graph. The error bars indicate s.d. (n = 2). Fzr-GFP^{fosmid} was expressed at the comparable level to endogenous Fzr in the *fzr-GFP^{fosmid}* larval brains. **c)** Anti-GFP immunoprecipitation using the larval brain extracts from wild type or the *fzr-GFP^{fosmid}* line. The input (left) and the precipitates (right) were analysed by immunoblotting against GFP and Spd2. Endogenous Spd2 was co-immunoprecipitated with Fzr-GFP^{fosmid}.

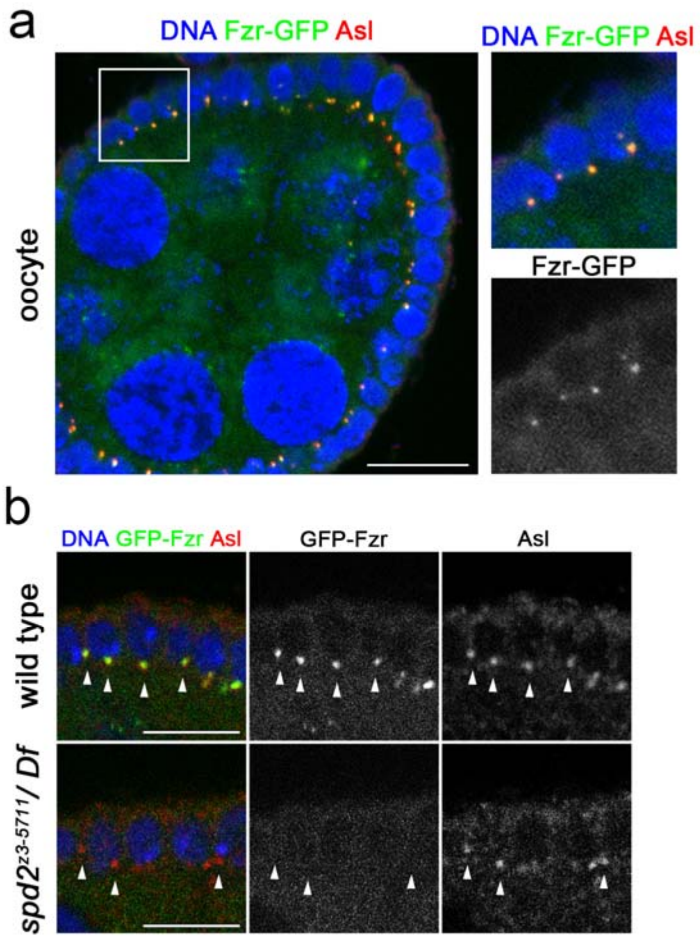

Supplementary figure 2. Fzr localises at the centrosome in a Spd2-dependent manner in ovarian follicle cells

a) An egg chamber expressing Fzr-GFP^{fosmid} (green) stained for the DNA (blue) and the centrosome marker Asl (red). In the panels to the right is the higher magnification of the follicle cells, showing Fzr-GFP^{fosmid} at the centrosome. **b)** Follicle cells of wild type and the *spd2*-null mutant (*spd2*$^{z3\text{-}5711}$*/Df(3L)BSC561*) expressing GFP-Fzr (green), stained for Asl (red) and DNA (blue). In the absence of Spd2, GFP-Fzr was not observed at the centrosomes. White arrowheads indicate the centrosomes. The scale bars represent 10 µm.

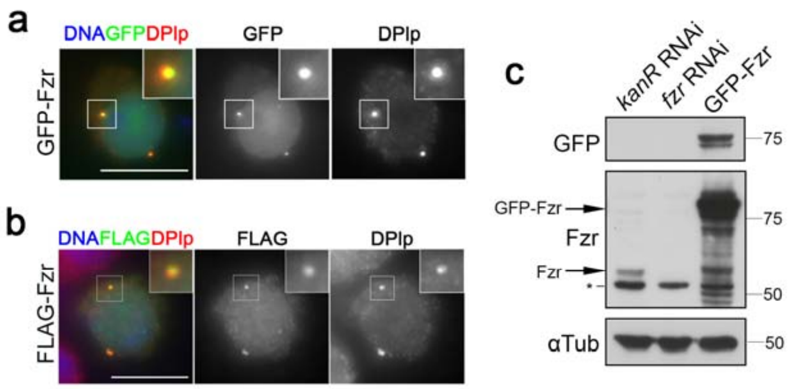

Supplementary figure 3. Both GFP- and FLAG-tagged Fzr localise at the centrosome in *D.mel-2* cells

a, b) *D.mel-2* cells transfected with the plasmids expressing GFP-Fzr (a) or FLAG-Fzr (b) and stained for DNA (blue) and the centrosome marker DPlp (red). Both GFP-Fzr (green in a) and FLAG-Fzr (green in b) localised at the centrosome, suggesting that the tags do not disturb the centrosomal localisation of Fzr. The higher magnification insets highlight the co-localisation of Fzr with DPlp at the centrosomes. The scale bars represent 10 µm. **c)** Immunoblotting of whole cell extracts from *D.mel-2* cells, treated with control *kanR* RNAi or *fzr* RNAi, or transfected with GFP-Fzr. Endogenous Fzr (indicated by an arrow) was efficiently depleted by *fzr* RNAi. The full length of GFP-Fzr (indicated by an arrow) was highly expressed in GFP-Fzr transfected cells.

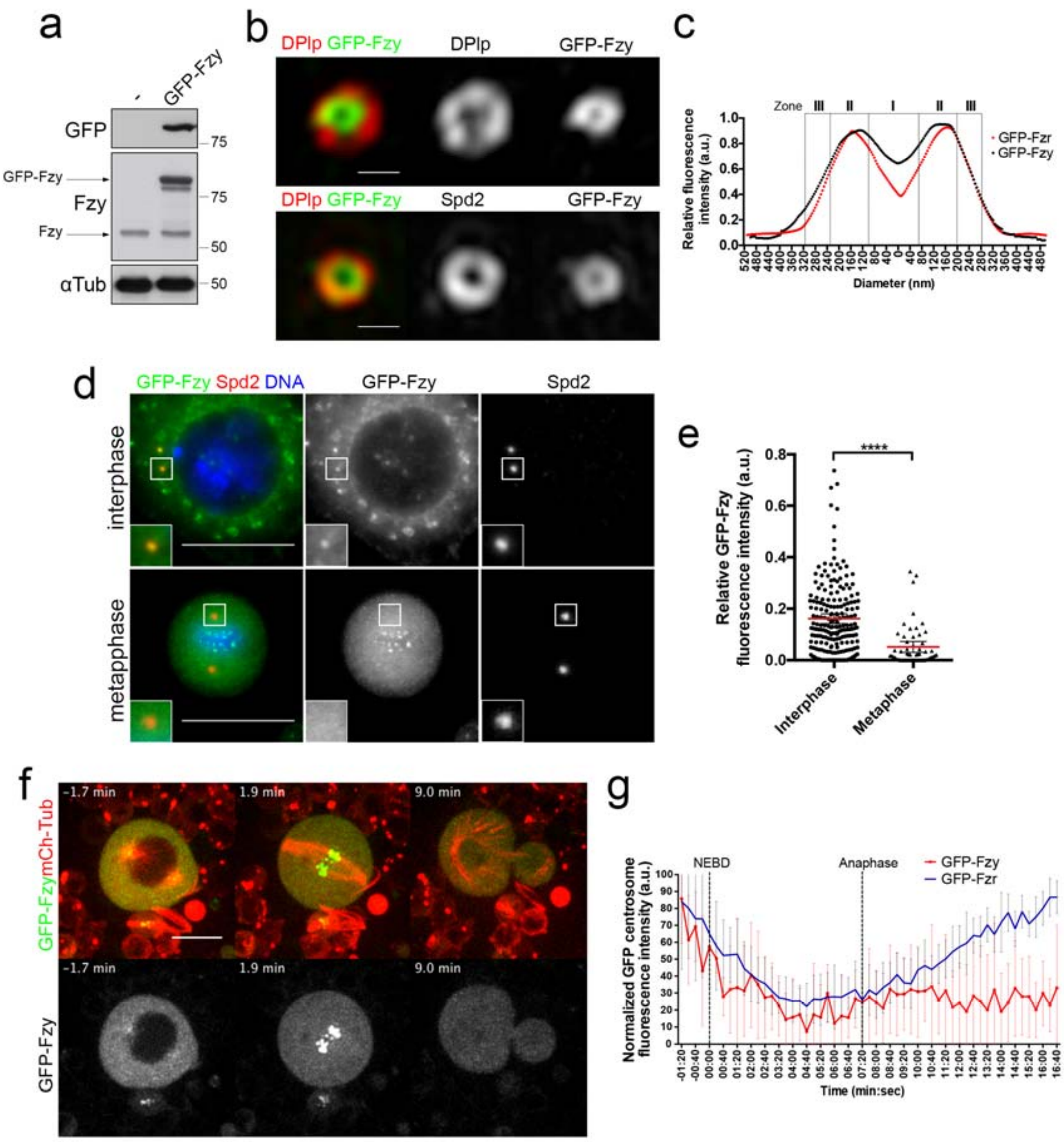

Supplementary figure 4. Fzy co-localises with Fzr at the inner centriole but shows different localisation dynamics

a) Immunoblotting of whole cell extracts from *D.mel-2* cells, untransfected (-) or transfected with the plasmid expressing GFP-Fzy. The arrows indicate the bands corresponding to endogenous Fzy and GFP-Fzy. **b)** 3D-SIM images of interphase centrosomes in *D.mel-2* cells expressing GFP-Fzy (green) stained for DPlp or Spd2 (red). GFP-Fzy co-localised with Spd2 at Zone II. **c)** Distribution curves of the signal intensities of GFP-Fzy and GFP-Fzr along the diameter of the centriole. The x-axis indicates the distance from the centre of the centriole (nm). GFP-Fzr and GFP-Fzy show a similar distribution. **d**) *D.mel-2* cells expressing GFP-Fzy (green) stained for DNA (blue) and Spd2 (red). GFP-Fzy co-localised with Spd2 at the centrosome during interphase (top panels) but not during mitosis (bottom panels). The high magnification

insets highlight the centrosomal localisation of GFP-Fzy. **e)** Quantification of the intensities of the centrosomal GFP-Fzy signal in interphase and mitotic cells. The result is presented in the dot plot (n = 200 or 56 in interphase or metaphase cells, respectively). The red bars indicate the means and the error bars the 95% confidence intervals (CIs). Significantly more GFP-Fzy accumulated at the centrosome during interphase than during mitosis (****, $p<0.0001$, Mann-Whitney *U* test). **f)** Tiles of selected still images from time-lapse live imaging of *Drosophila* NBs co-expressing GFP-Fzy (green) and mCherry-Tubulin driven by wor-Gal4 driver (red). GFP-Fzy was detectable at the centrosomes as well as in the cytoplasm until prophase (-1.7 min). After NEBD, GFP-Fzy dissociated from the centrosome and highly accumulated at kinetochores during prometaphase (1.9 min). GFP-Fzy did not return to the centrosome until late telophase. **g)** Centrosomal GFP-Fzy fluorescence intensity was measured in live NBs and the mean values are presented in the line graph (red line, n = 8). The error bars indicate s.d. The data of GFP-Fzr used in Fig. 2e is also shown for comparison (blue line). GFP-Fzy and GFP-Fzr dissociate from the centrosome at a similar timing during mitosis. However, GFP-Fzy remains dissociated for a longer period during mitotic exit. Scale bars, 30 nm (b), 10 µm (d) and 5 µm (f).

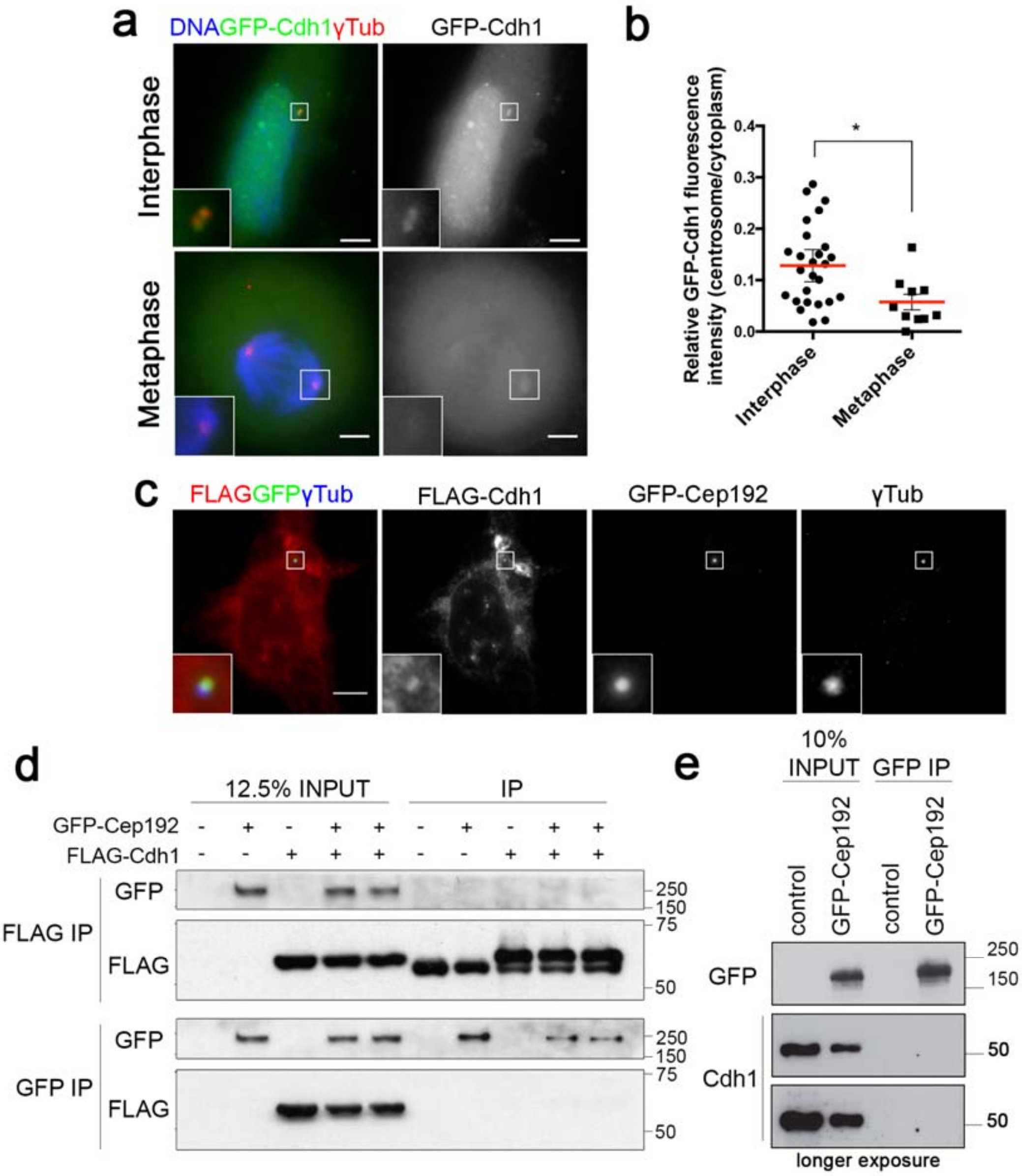

Supplementary figure 5. **Cdh1 localises at the centrosome, but does not interact with Cep192**

a) HeLa cells expressing GFP-tagged Cdh1 (green) stained for DNA (blue) and a centrosomal marker γ–Tubulin (γTub, red). GFP-Cdh1 showed clear centrosomal localisation during interphase (top) but not during mitosis (bottom) in HeLa cells. The centrosomes are highlighted in the high magnification insets. The scale bars indicate 10 µm. **b)** The centrosomal signal intensity of GFP-Cdh1 was quantified in interphase or metaphase HeLa cells expressing GFP-Cdh1(n = 26 or 10, respectively) and is presented in a dot blot. The red bars indicate the mean values and the error bars the 95% CIs. More GFP-Cdh1 accumulated at centrosomes during interphase than during mitosis (*, p = 0.0110, unpaired *t*-test). **c)** HeLa cells co-transfected with GFP-tagged Cep192 (green) and FLAG-tagged Cdh1, stained for FLAG (red) and γTub (blue).

FLAG-Cdh1 and GFP-Cep192 co-localised with γTub at centrosomes. The scale bar indicates 10 μm. **d)** Reciprocal anti-GFP or anti-FLAG immunoprecipitation from HeLa cells transfected with FLAG-Cdh1 and GFP-Cep192. FLAG-Cdh1 and GFP-Cep192 did not interact with each other in the co-immunoprecipitation assays. **e)** Anti-GFP immunoprecipitation from HeLa cells expressing GFP-Cep192. The input and the precipitates were analysed with anti-GFP and anti-Cdh1 antibodies. Endogenous Cdh1 was not co-precipitated with GFP-Cep192.

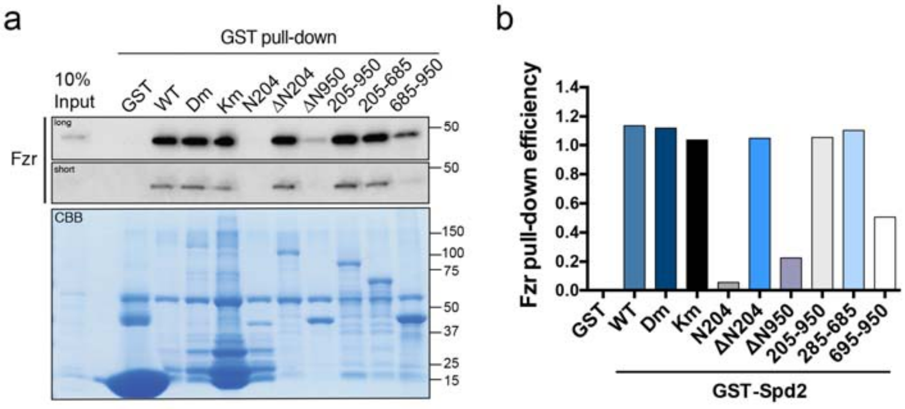

Supplementary figure 6. The 205-685 region of Spd2 directly interacts with Fzr

a) The *in vitro* binding assay using purified GST or the truncated or mutated forms of GST-tagged Spd2 immobilised on Glutathione Sepharose beads, alongside ^{35}S-labelled His-tagged Fzr. The input and the bound fractions were analysed by autoradiograph and Coomassie Blue staining. **b)** The Fzr pull down efficiencies were quantified for individual baits and are presented in the bar graph. The Spd2 fragments containing the wild type 205-685 region showed higher affinity for Fzr compared to the fragments lacking this region.

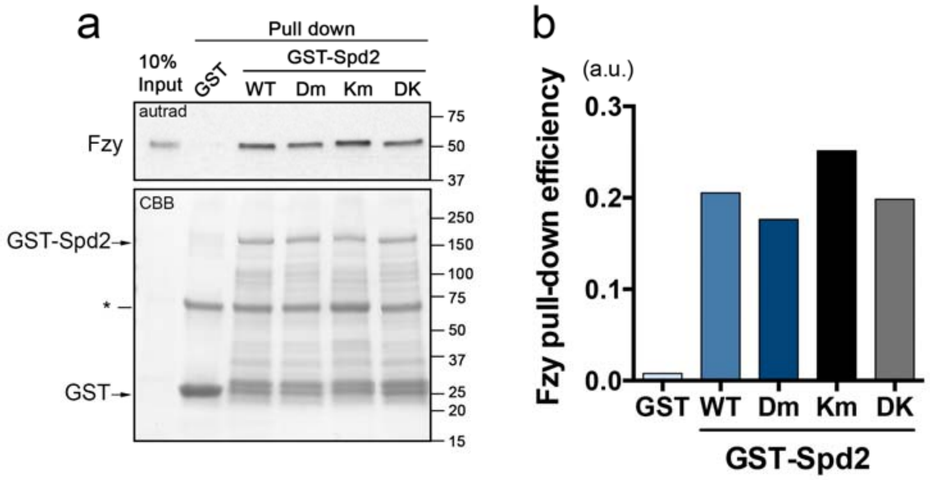

Supplementary figure 7. The D-box or KEN-box mutation in Spd2 does not affect the interaction with Fzy

a) The *in vitro* binding assay using purified GST or GST-fused Spd2-WT, Dm, Km and DK alongside ^{35}S-labelled Fzy. The input and the bound fractions were analysed by autoradiograph and Coomassie Blue staining. **b)** The Fzy pull down efficiencies for individual baits were quantified and are shown in the bar graph. Neither the D-box nor KEN-box mutations in Spd2 significantly affected the interaction between Spd2 and Fzy *in vitro*.

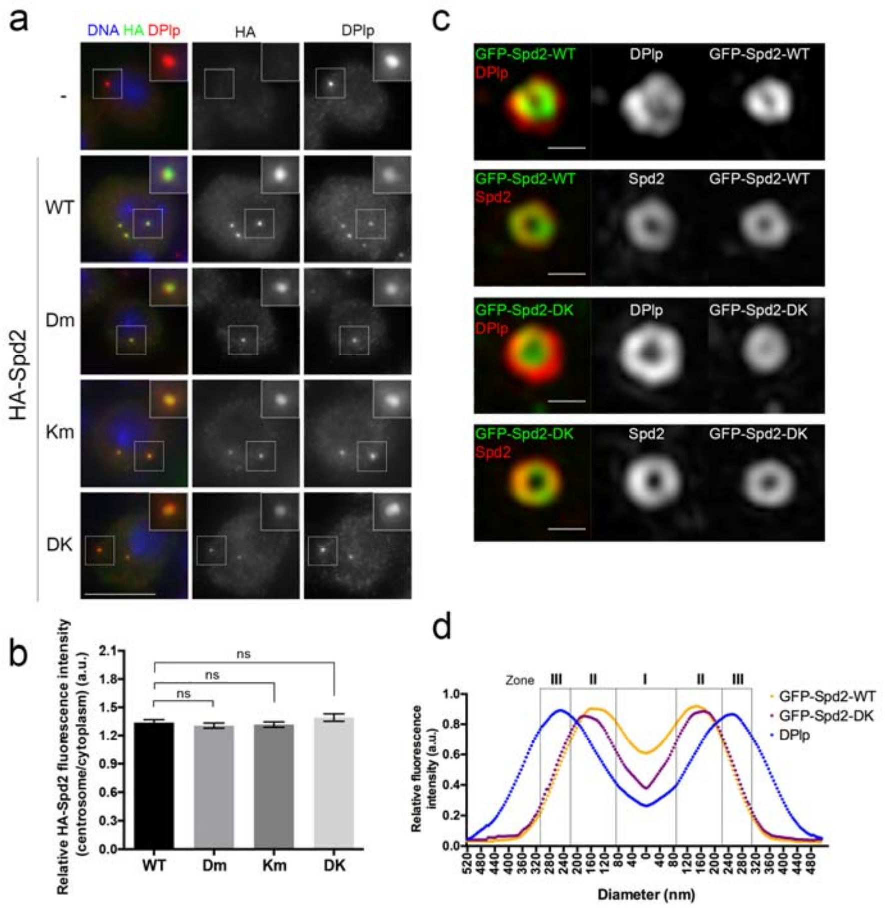

Supplementary figure 8. The D-box or KEN-box mutations in Spd2 do not alter the centrosome localisation of Spd2

a) *D.mel-2* cells untransfected or transfected with the plasmids expressing HA-Spd2-WT, Dm, Km or DK (green), stained for DNA (blue) and DPlp (red). All of the Spd2 constructs co-localised with the centrosome marker Dplp. The insets show the higher magnification of Spd2-Dplp co-localization. Scale bars, 10 µm. **b)** The centrosomal signal intensities of HA-Spd2 were quantified and are shown in a bar graph (n = 100). The heights of the bars indicate the mean values and the error bars the 95% CIs. All the mutant forms of HA-Spd2 accumulated at the centrosome at levels comparable to HA-Spd2-WT (ns, not significant, $p>0.05$, Mann-Whitney *U* test). **c)** 3D-SIM super-resolution images of interphase centrosomes in *D.mel-2* cells expressing either GFP-Spd2-WT or GFP-Spd2-DK (green), stained for DPlp or Spd2 (red). GFP-Sod2-WT and GFP-Spd2-DK co-localised with endogenous Spd2 at the inner region of the centriole within a ring formed by DPlp. The scale bars correspond to 300 nm. **d)** Distribution curves of the signal intensities of GFP-Spd2-WT (yellow line), GFP-Spd2-DK (black line) and DPlp (blue line) along the diameter of the centriole. GFP-Spd2-WT and GFP-Spd2-DK show overlapping peaks and are localised at Zone II.

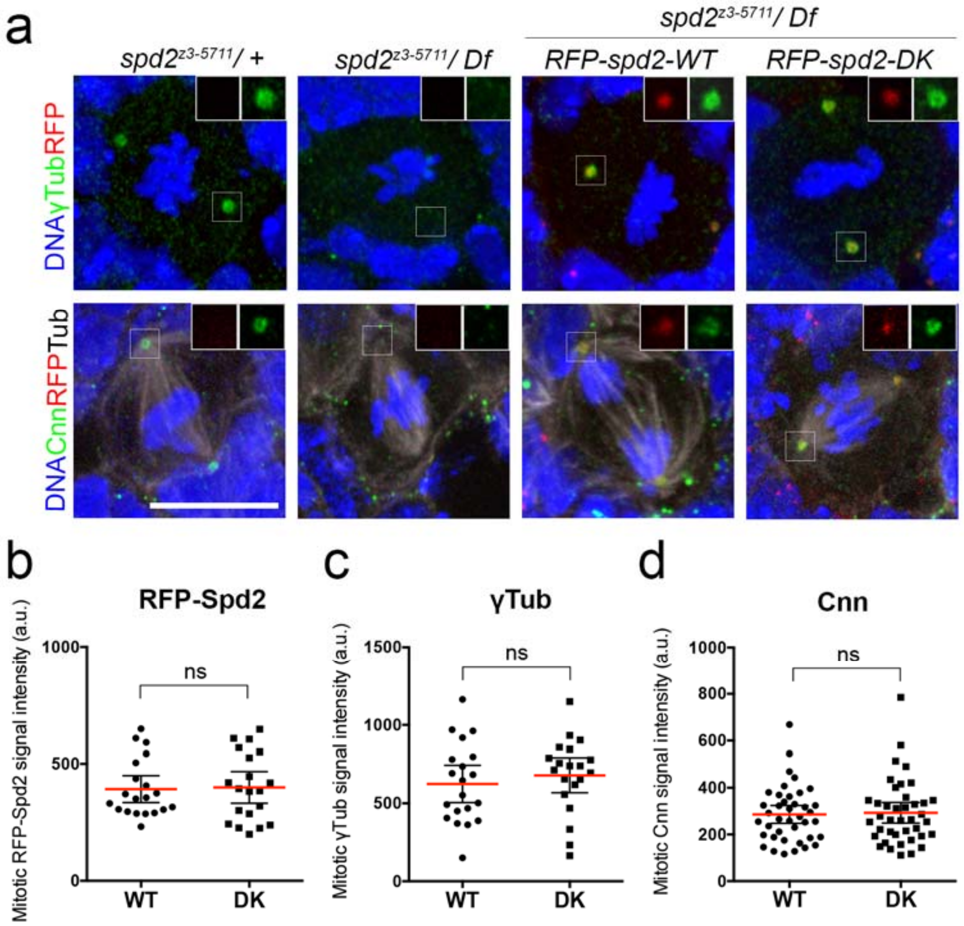

Supplementary figure 9. The D-box or KEN-box mutations in Spd2 can accumulate PCM

a) Images of larval NBs of the control (*spd2*$^{z3\text{-}5711}$/+), the *spd2*-null mutant (*spd2*$^{z3\text{-}5711}$*/Df(3L)BSC561*), and the Spd2-WT or Spd2-DK rescued larvae stained for DNA (blue), and γTub (green, top panels), or Centrosomin (Cnn, green, bottom panels) and α-Tubulin (white, bottom panels). The control NBs recruit γTub and Cnn to the centrosomes during mitosis, whereas the *spd2*-null mutant NBs fail to recruit these PCM components. RFP-Spd2-WT and RFP-Spd2-DK (red) localise at the centrosomes at comparable levels and can restore Cnn and γTub recruitment during mitosis in the *spd2*-null mutant NBs, exhibiting microtubule focusing at spindle poles. The insets show higher magnification of the centrosomes. Scale bars, 10 µm. **b-d)** Quantification of the centrosomal signal intensities of RFP-Spd2 (b), γTub (c) and Cnn (d) during mitosis (metaphase) in the Spd2-WT or Spd2-DK rescued NBs. The quantification results were shown in the dot blots. The red bars indicate means and error bars the 95% CIs. RFP-Spd2-WT and RFP-Spd2-DK localised at the centrosome at a similar intensity (n = 38 and 36) and recruited the comparable amounts of γTub and Cnn to the centrosome during mitosis (n = 20 and 40, respectively). ns, not significant. $p>0.05$, unpaired *t*-test.

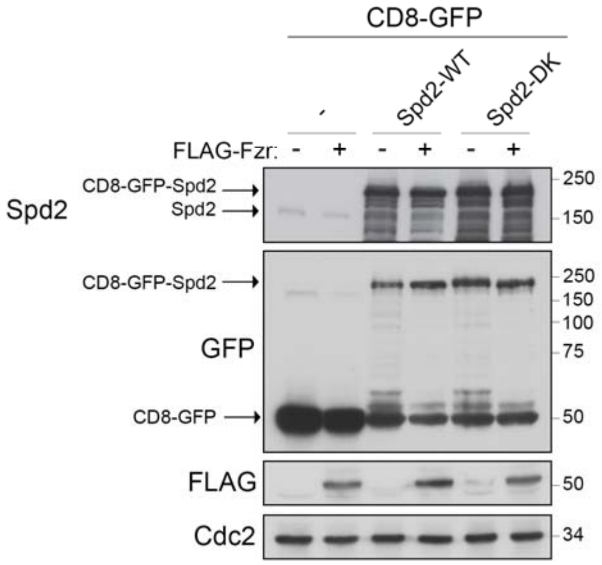

Supplementary figure 10. Confirmation of the expression of CD8-GFP-fused Spd2-WT or Spd2-DK in *D.mel-2* cells

Immunoblotting analysis of whole cell extracts from *D.mel-2* cells transfected with the plasmids expressing CD8-GFP, CD8-GFP-Spd2-WT or CD8-GFP-Spd2-DK, with or without the plasmid expressing FLAG-Fzr. CD8-GFP, CD8-GFP-Spd2-WT and CD8-GFP-Spd2-DK were detected at the expected size (indicated by the arrows).

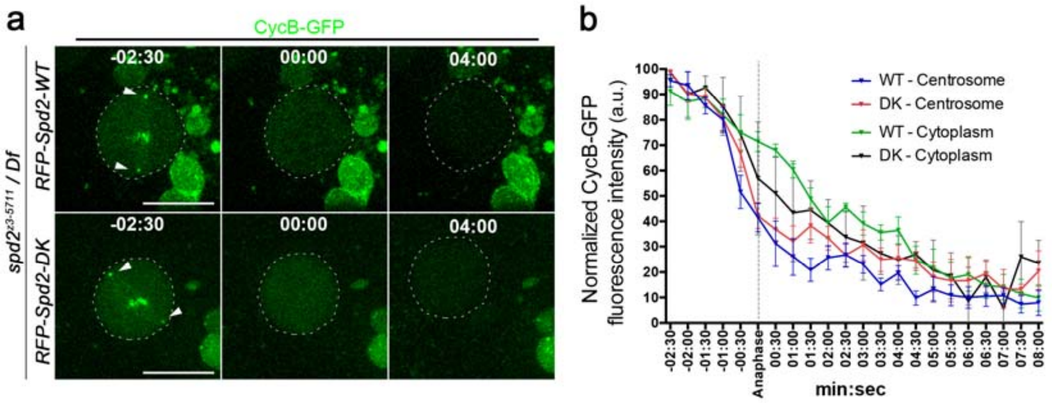

Supplementary figure 11. Centrosomal localisation of Fzr is not rate-limiting for Cyclin B degradation during mitotic exit in larval neuroblasts

a) Selected still images from time-lapse movies of the Spd2-WT or Spd2-DK rescued NBs expressing GFP-tagged Cyclin B (CycB-GFP, green). In both of the Spd2-WT and Spd2-DK rescued NBs, CycB-GFP shows similar dynamics during mitosis. In early mitosis CycB-GFP accumulates highly at the centrosome and on mitotic spindle, and weakly in cytoplasm (-02:30). Upon anaphase onset (00:00), CycB-GFP started to degrade at the centrosomes and rapidly disappeared from spindles. Subsequently, the cytoplasmic CycB-GFP signal gradually disappeared (04:00). White arrowheads indicate the centrosomes, and white dotted lines outline the neuroblasts. Scale bars: 10 µm. **b)** Biphasic degradation kinetics of CycB-GFP during mitotic exit in the Spd2-WT and Spd2-DK rescued NBs. The centrosomal and cytoplasmic signal intensities of CycB-GFP were separately measured at each time point during mitotic exit and the mean value of the normalised signal intensities was plotted in the line graph (Spd2-WT rescued NBs, n = 8; Spd2-DK rescued NBs, n = 7; error bars, s.d. The Spd2-WT and Spd2-DK rescued NBs showed similar degradation kinetics of the cytoplasmic and centrosomal CycB-GFP signal. The vertical dotted line indicates the time of anaphase onset.

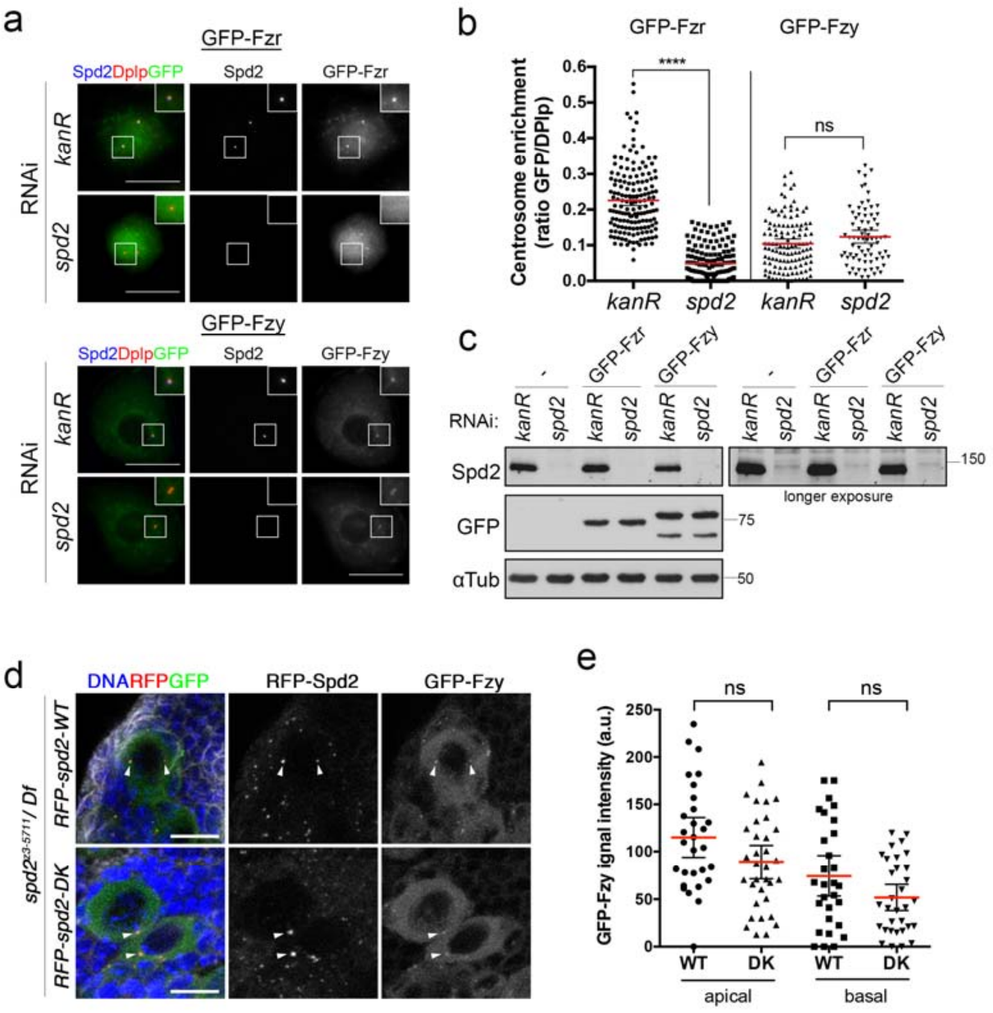

Supplementary figure 12. The centrosomal localisation of the mitotic APC/C activator Fzy is not dependent on Spd2

a) *D.mel-2* cells stably expressing GFP-Fzy (green, bottom and second to bottom panels) or GFP-Fzr (green, top and second to top panels) were transfected with the control *kanR* dsRNA or *spd2* dsRNA and stained for DPlp (red), and Spd2 (blue). Upon *spd2* RNAi, endogenous Spd2 became undetectable. Whilst centrosomal GFP-Fzr was lost by *spd2 RNAi*, centrosomal GFP-Fzy appeared unaffected. The scale bar represents 10 µm. **b)** The centrosomal GFP signal was quantified in the cells used in a and individual measurements were presented in a dot plot (n≥75). The horizontal red lines indicate the mean values and the error bars the 95% CIs. The centrosomal GFP-Fzr signal significantly decreased upon Spd2 depletion (****, p<0.0001, Mann-Whitney *U* test), whilst the centrosomal GFP-Fzy signal was unaffected (ns, not significant, p>0.05, Mann-Whitney *U* test). **c)** Western blotting of the whole cell extracts used in **a** and **b**. Endogenous Spd2 was efficiently depleted upon *spd2* RNAi and both GFP-Fzy and GFP-Fzr were expressed at comparable levels. **d)** The Spd2-WT rescued and Spd2-DK rescued

NBs expressing GFP-Fzy (green) were stained for DNA (blue). GFP-Fzy co-localised with RFP-Spd2 (red) in both rescue NBs. The scale bar represents 10 μm. The arrows point to the centrosomes. **e)** Quantification of the centrosomal signal of GFP-Fzy in the interphase Spd2-WT rescued and Spd2-DK rescued NBs. Centrosomal GFP-Fzy was comparable between the Spd2-WT rescued and Spd2-DK rescued NBs at the apical centrosomes and the basal centrosomes, respectively (n = 29, 34; ns, not significant, $p>0.05$, unpaired *t*-test). Red bars indicate the means. Error bars, 95% CIs.

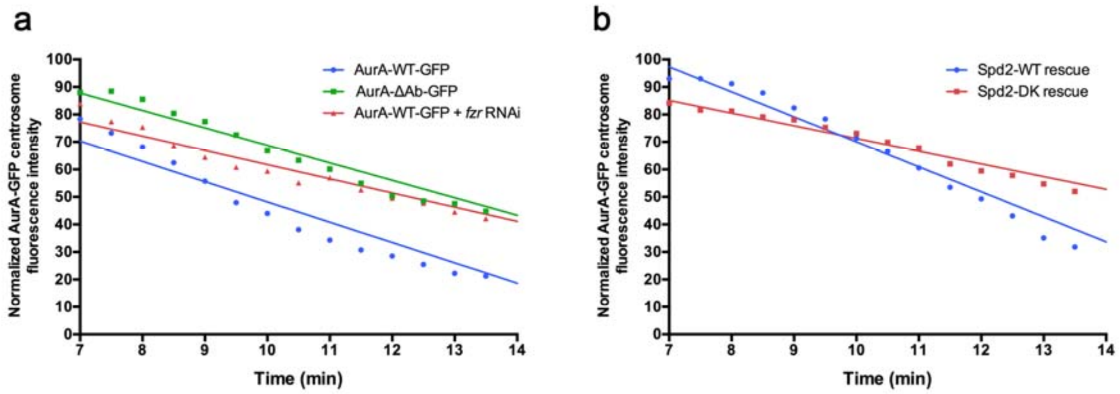

	AurA-WT-GFP	AurA-ΔAb-GFP	AurA-WT-GFP + *fzr* RNAi
Slope	-7.372 ± 0.7782	-6.349 ± 0.4832	-5.160 ± 0.4044

	Spd2-WT rescue	Spd2-DK rescue
Slope	-9.091 ± 0.5485	-4.621 ± 0.2560

Supplementary figure 13. Linear regression of AurA degradation kinetics

a) Linear regression of AurA degradation kinetics in NBs of the indicated genotypes. The mean values of the AurA-GFP signal between the onset and the completion of AurA-WT-GFP degradation in control NBs (7 min and 14 min after Anaphase onset, respectively) were plotted and the best-fit lines are shown. AurA-WT-GFP (blue line) showed the degradation rate (K^{deg}) of 7.372 ± 0.7782 % min^{-1} (the values after ± indicate standard errors), whilst the AurA-ΔAb (green line) and *fzr RNAi* (red line) showed slower degradation rates (K^{deg} = 6.349 ± 0.4832 % min^{-1} and 5.160 ± 0.4044 % min^{-1}, respectively). **b)** Linear regression of AurA-GFP degradation kinetics in the Spd2-WT rescued and Spd2-DK rescued NBs. The degradation rate of AurA-GFP is much slower in the Spd2-DK rescued NBs (red line, K^{deg} = 4.621 ± 0.2560 % min^{-1}), compared to that in the Spd2-WT rescued NBs (blue line, K^{deg} = 9.091 ± 0.5485 % min^{-1}. The values after ± indicate the standard errors).

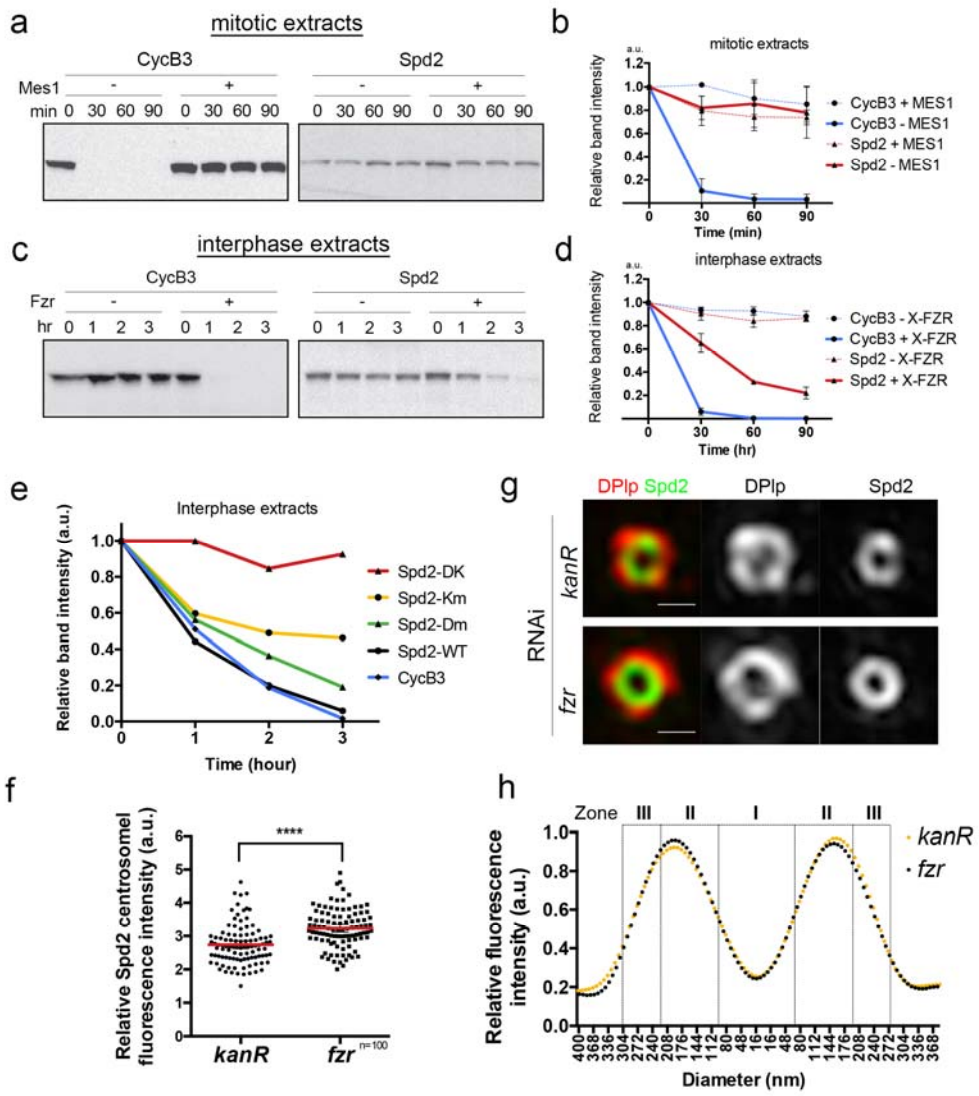

Supplementary figure 14. Spd2 is an APC/C^{Fzr} substrate

a-d) The in *vitro* APC/C-dependent destruction assay using ^{35}S-labeled *Drosophila* Cyclin B3 (CycB3) and Spd2 as substrates in mitotic (a, b) and interphase (c, d) frog egg extracts. CycB3 was rapidly degraded upon APC/C activation both in mitotic and interphase egg extracts, whilst Spd2 was only degraded in interphase extracts. The signal intensities of CycB3 and Spd2 bands in the autoradiograph from the *in vitro* destruction assay were quantified and the mean intensities were plotted in the line graph (b, d, n = 3). Error bars, s.d. Spd2 rapidly degrade in interphase egg extracts (c, d), but not in mitotic extracts (a, b). **e**) The signal intensities of the bands of CycB3 and the various forms of Spd2 in the destruction assay (Fig. 8a) were quantified and the mean intensities were plotted in the line graph. The D-box and KEN-box mutation stabilises Spd2 in interphase egg extracts. Spd2-DK is completely stable. **f)** The centrosomal signal intensity of endogenous Spd2 in *D.mel*-2 cells transfected with *kanR* dsRNA or *fzr* dsRNA. The Spd2 centrosomal signal intensity was measured and the individual measurements are presented in a dot plot (n = 100). The horizontal red bars indicate the mean values and the

error bars the 95% CIs. Spd2 accumulated at the centrosomes upon Fzr depletion (****, $p<0.0001$, unpaired *t*-test). **g)** 3D-SIM super-resolution images of interphase centrosomes in *D.mel-2* cells transfected with *kanR* dsRNA or *fzr* dsRNA, stained for DPlp (red) and Spd2 (green). Fzr depletion did not affect the Zone II localisation of Spd2. The scale bars correspond to 500 nm. **h)** Distribution curves of the signal intensities of Spd2 upon control *kanR* RNAi (yellow curve) and *fzr* RNAi (black curve). Although Spd2 accumulates at the centrosome upon *fzr* RNAi (f**),** the distribution pattern of Spd2 within a centrosome did not change.

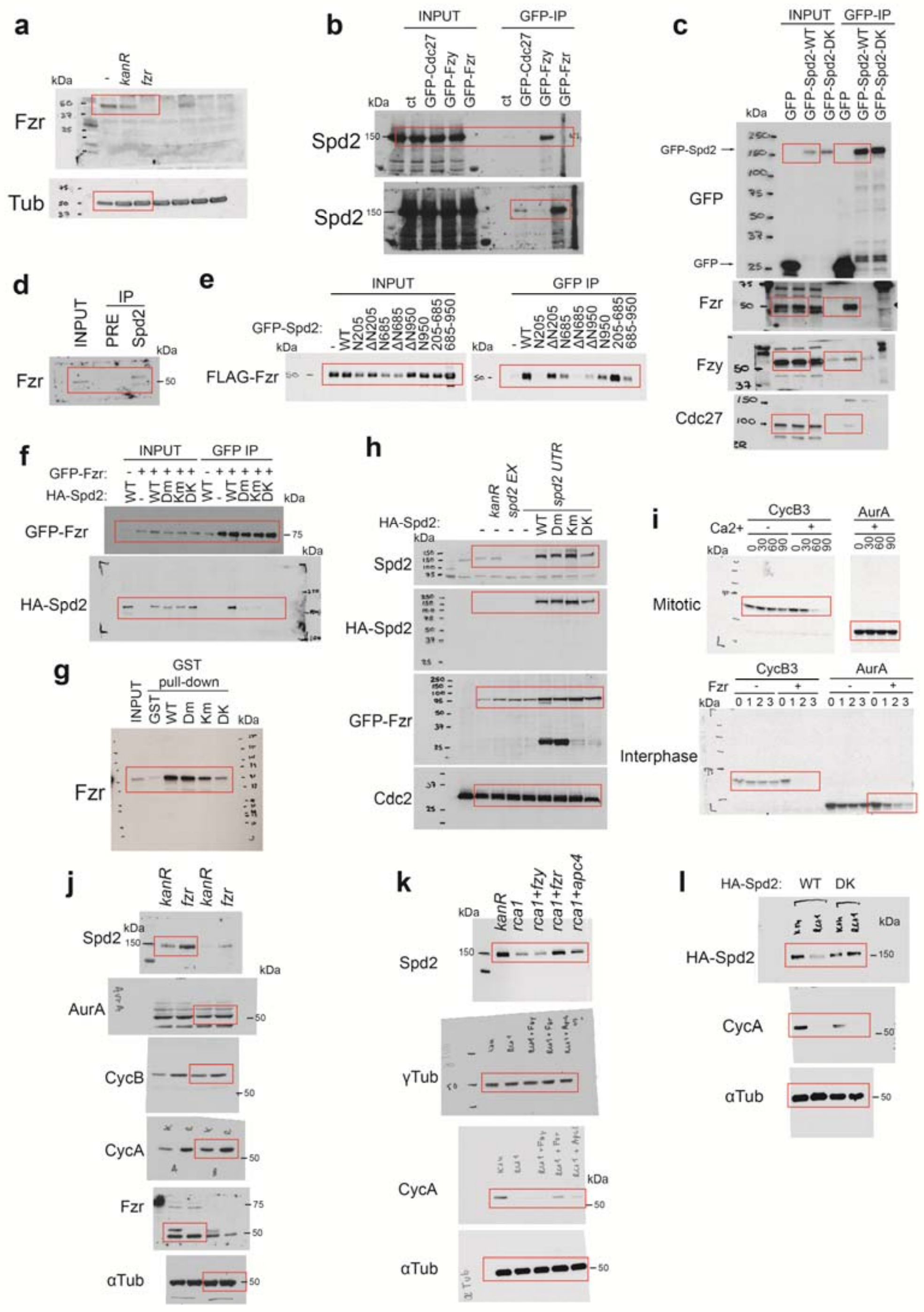

Supplementary figure 15. The full sized immunoblots or autoradiographs used in this study

The whole western blots, part of which are presented in the main figures, are shown. A single membrane was often cut to allow detection of two or more different antigens. The antibodies used are indicated next to the blots and the red rectangles indicate the portions cropped for the main figures. **a**) Whole western blots used in Figure 1e. Fzr (~53 kDa, top panel) and α-Tubulin (~50 kDa, bottom panel) were detected. **b**) Anti-Spd2 blots used in Figure 3c. The bottom panel represents a longer exposure of the same membrane. **c**) Whole western blots used in Figure 3e.

GFP-Spd2 (~150 kDa), the GFP tag alone (~27 kDa), Fzr (~53 kDa), Fzy (~57 kDa, third panel) and Cdc27 (~100 kDa) were detected. **d**) Whole anit-Fzr (~53 kDa) blot used in Figure 3g. **e**) anti-FLAG blot used in Figure 4b. FLAG-Fzr (54 kDa) was detected. **f**) Anti-GFP blot (top panel) and anti-HA blot (bottom panel) used in Figure 4f. GFP-Fzr (~75 kDa) and HA-Spd2 (~130 kDa) were detected. **g**) Whole autoradiograph used in Figure 4g. Radiolabelled Fzr (~53kDa) was detected. **h**) Whole blots used in Figure 5a. Spd2 (~130 kDa), HA-Spd2 (~130 kDa), GFP-Fzr (~75 kDa) and Cdc2 (~37 kDa) were detected. **i**) Whole autoradiographs of mitotic (top panels) and interphase (bottom panels) destruction used in Figure 6a. Radiolabeled CycB3 (~65 kDa) and AurA (~46kDa) were detected. **j**) Whole blotsuse in Figure 8c. Spd2 (~130 kDa), AurA (~46 kDa), CycB (~60 kDa), CycA (~56 kDa), Fzr (~53 kDa) and α-Tubulin (~50 kDa) were detected. The experiment was performed in duplicates, indicated as A and B. **k**) Whole blots used in Figure 8d. Spd2 (~130 kDa), γ-Tubulin (~53 kDa), CycA (~56 kDa), and α-Tubulin (~50 kDa) were detected. **i**) Whole blots used in Figure 8e. HA-Spd2-WT or HA-Spd2-DK (~130 kDa), CycA (~56 kDa), and α-Tubulin (~50 kDa) were detected.
